# Imaging extracellular ATP with a genetically-encoded, ratiometric fluorescent sensor

**DOI:** 10.1371/journal.pone.0187481

**Published:** 2017-11-09

**Authors:** Jason M. Conley, Saranya Radhakrishnan, Stephen A. Valentino, Mathew Tantama

**Affiliations:** 1 Department of Chemistry, Purdue University, West Lafayette, Indiana, United States of America; 2 Purdue Institute for Integrative Neuroscience, Purdue University, West Lafayette, Indiana, United States of America; 3 Purdue Interdisciplinary Life Science Graduate Program, Purdue University, West Lafayette, Indiana, United States of America; 4 Purdue Institute for Inflammation, Immunology, & Infectious Disease, Purdue University, West Lafayette, Indiana, United States of America; University Hospital Freiburg, GERMANY

## Abstract

Extracellular adenosine triphosphate (ATP) is a key purinergic signal that mediates cell-to-cell communication both within and between organ systems. We address the need for a robust and minimally invasive approach to measuring extracellular ATP by re-engineering the ATeam ATP sensor to be expressed on the cell surface. Using this approach, we image real-time changes in extracellular ATP levels with a sensor that is fully genetically-encoded and does not require an exogenous substrate. In addition, the sensor is ratiometric to allow for reliable quantitation of extracellular ATP fluxes. Using live-cell microscopy, we characterize sensor performance when expressed on cultured Neuro2A cells, and we measure both stimulated release of ATP and its clearance by ectonucleotidases. Thus, this proof-of-principle demonstrates a first-generation sensor to report extracellular ATP dynamics that may be useful for studying purinergic signaling in living specimens.

## Introduction

Extracellular adenosine triphosphate (ATP) is a central component of purinergic signaling, and purinergic signaling contributes to a vast array of fundamental physiological processes and pathophysiological conditions including neuron-glia communication[1–3], immune responses[4, 5], inflammation[6, 7], and cancer[8–10]. ATP is released from cells into the extracellular space by a variety of mechanisms such as stimulated exocytosis and conductive passage through hemichannels[11–14]. Following release, extracellular ATP directly modulates purinergic receptors in an autocrine and paracrine manner[15]. For example, in an immune context ATP leakage from apoptotic cells in healthy tissue[13, 16] or from damaged cells in injured tissue[17–19] acts as a chemotactic signal for clearance by phagocytes. In the context of nervous tissue, ATP released by astrocytes[20] can regulate synaptic and network excitability[21–25]. Furthermore, extracellular ATP is central to purinergic signaling not only because of its direct effects but also because extracellular ectonucleotidases, such as CD39[10] and CD73[26], hydrolyze it to the additional purinergic signaling molecules ADP, AMP, and adenosine[27]. Depending on the physiological context[28, 29], these metabolites uniquely modulate distinct sets of ATP-gated ionotropic P2X receptors, ATP and ADP-modulated metabotropic P2Y receptors, and P1 adenosine receptors[30–32]. It is important to understand extracellular ATP dynamics as a fundamental aspect of physiology and because ATP-dependent receptors, as well as the ATP release and clearance machinery, are potential therapeutic targets[33, 34]. However, deficiencies in our understanding of the broad concentration ranges, timescales, and distances over which extracellular ATP acts currently obscures the roles of purinergic signaling in both healthy and diseased tissue. Therefore, in order to establish a clear picture of purinergic signaling in physiology, it is necessary to distinguish the role of extracellular ATP from its hydrolysis products and to quantitatively measure extracellular ATP dynamics directly.

Direct measurements of extracellular ATP employ diverse techniques including biochemical endpoint assays, microelectrode sensors, and fluorescent ATP analogues[15, 35]. In particular, membrane-tethered luciferase continues to provide critical new knowledge about purinergic signaling in cancer biology, immunology, and beyond[36–42]. These methods have yielded invaluable insight into ATP signaling; however, new methods are needed to push beyond the current limitations in spatial and temporal resolution. These limitations prevent the precise understanding of changes in extracellular ATP levels that occur within seconds and minutes at cellular and subcellular length scales. For example, current techniques are limited in their applications to complex tissue because they require chemical additives, damage tissue with an invasive probe, or consume ATP upon measurement. More recently, genetically-encoded fluorescent protein-based sensors have been developed as relatively non-invasive tools with high spatiotemporal resolution to study *intracellular* ATP. These include the ATeam family of sensors that report intracellular ATP dynamics by a change in Förster resonance energy transfer (FRET) between two fluorescent proteins[43], and the QUEEN[44] and Perceval[45, 46] sensors that use a single circularly-permuted fluorescent protein. Though exploited in a number of intracellular contexts, these sensors have not been used to detect extracellular ATP.

Here, we re-engineer a ratiometric ATeam FRET-based ATP sensor by targeting it to the cell surface, and report its use as a genetically-encoded fluorescent sensor of extracellular ATP. We report its design, characterization, and proof-of-principle that it can be used to image and monitor real-time changes in extracellular ATP levels caused by endogenous clearance and release mechanisms in cell culture, using Neuro2A cells as a principal test platform for the sensor.

## Results

### Sensor construction and characterization

**Fig 1:**
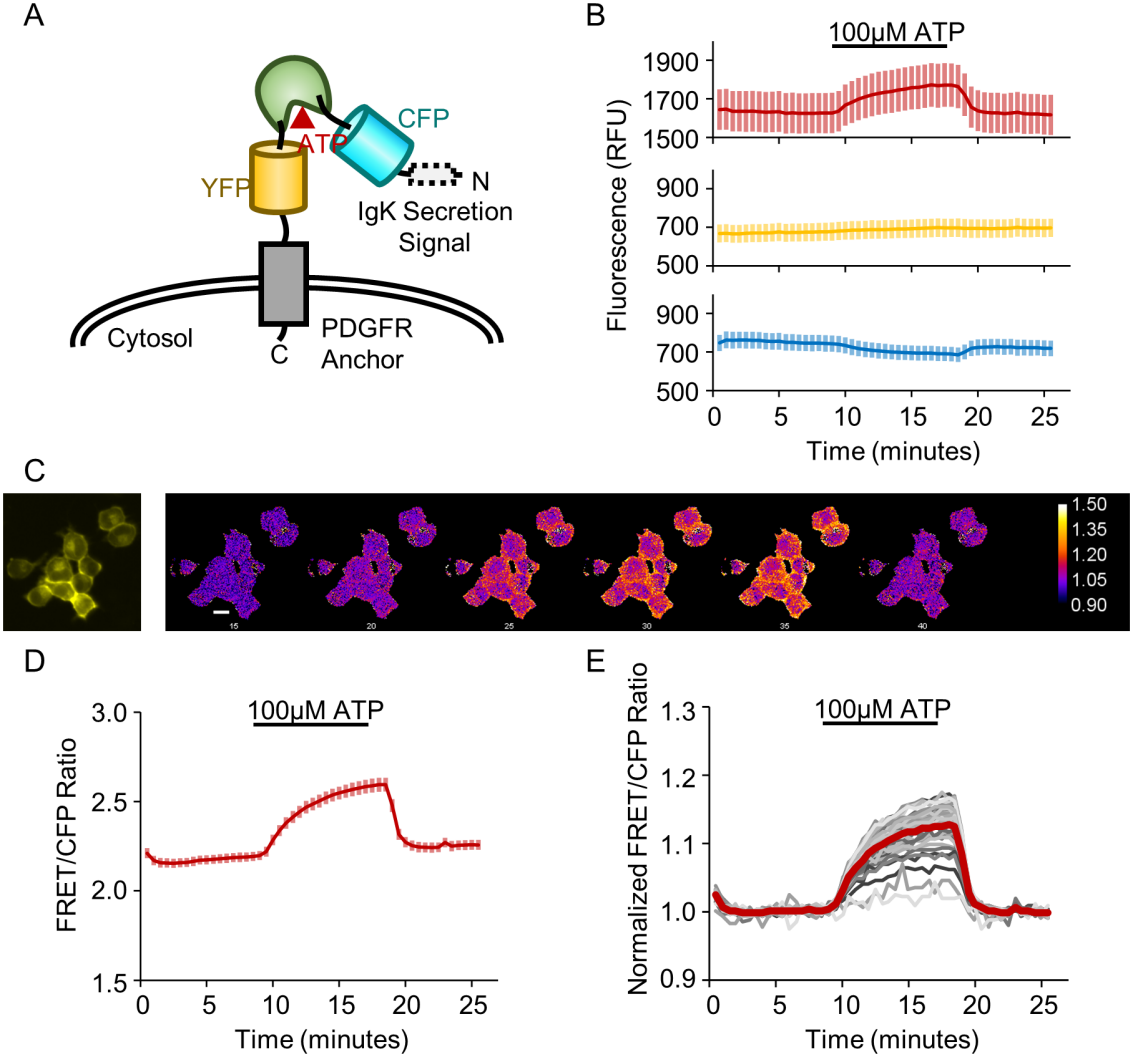
The ecATeam3.10 sensor detects extracellular ATP. (A) Schematic of ecAT3.10 design in which the ATeam3.10 FRET-based ATP sensor is displayed on the cell surface via a PDGFR transmembrane anchor. (B) Fluorescence intensity in the FRET channel increases upon wash-in of 100 μM ATP while the CFP donor intensity decreases. As expected, the YFP direct acceptor channel does not show an ATP-dependent change. Cells were imaged under continuous perfusion. (C) Representative widefield fluorescence images for the cells analyzed in (D-E). The first panel in the YFP channel shows morphology, the subsequent panels are false colored to show the change in the normalized FRET/CFP pixel-by-pixel ratio signal. Scale bar is 20μm. (D) The average FRET/CFP emission ratio, which we refer to as the ratio signal, shows a robust increase of 0.27 ± 0.01 (n = 41 cells). Values and solid line traces are cell means, and errors and error bars are standard errors of the means. (E) To account for drift, a linear baseline was fitted, and the ratio signal was normalized on a cell-by-cell basis (individual cells, gray), showing an average fold change of 1.127 ± 0.005 (population average, red).

To generate a sensor of extracellular ATP, we targeted a soluble ATeam ATP sensor to the cell surface. The ATeam family of sensors, first developed by Imamura and co-workers, are generally composed of an ε subunit from a bacterial F_O_F_1_-ATP synthase that is fused between a cyan fluorescent protein (CFP) and a yellow fluorescent protein (YFP)[43]. ATP binding induces a conformational change that increases Förster-type resonance energy transfer (FRET) between the CFP donor and YFP acceptor[43]. The soluble ATeam3.10 sensor was chosen as a starting point because it can provide a ratiometric fluorescence readout, which facilitates quantitative and longitudinal live-cell imaging studies by normalizing for expression level and decreasing signal drift. It is also a higher-affinity variant that better matches the estimated physiological range of extracellular ATP. In order to target the soluble ATeam3.10 sensor to the cell surface, we employed a strategy previously used to engineer extracellular glutamate sensors[47–49]. The ATeam3.10 sensor was fused between an IgK leader sequence[50] on the N-terminus and a transmembrane anchor domain from the platelet-derived growth factor receptor (PDGFR)[51] on the C-terminus. These modifications direct the sensor to the secretory pathway and tether the sensor to the extracellular face of the plasma membrane, respectively (Fig 1). The resulting membrane-tethered sensor was termed ecAT3.10 (extracellular ATeam3.10).

Initially, the ecAT3.10 sensor was expressed in cultured mammalian cells to test for expression at the cell surface and for a response to exogenous ATP. Even in widefield imaging mode, the fluorescence signals were clearly localized to the cell perimeter of Neuro2A cells, indicating successful targeting of ecAT3.10 to the plasma membrane (Fig 1). Furthermore, under continuous bath perfusion conditions, wash-in of 100 μM ATP resulted in an increase in FRET emission intensity and a decrease in CFP donor fluorescence intensity, which is consistent with the expected increase in FRET upon ATP binding (Fig 1 and S1 Fig). At the cell population level, the ecAT3.10 FRET/CFP emission ratio signal (referred to as the “ratio signal”) reached a maximal increase of 0.27 ± 0.01 (Fig 1), representing a peak fold-increase of 1.127 ± 0.005 (mean ± sem, n = 41 cells) in the presence of ATP relative to the ratio signal in the absence of ATP (Fig 1D). Upon washout of extracellular ATP from the bath, the ecAT3.10 ratio signal returned to baseline, demonstrating that the sensor can be used to reversibly detect changes in extracellular ATP. Interestingly, ecAT3.10 was able to detect significant cell-to-cell variability in the response magnitude (Fig 1E), which may be related to physical characteristics of the cell surface or endogenous activity under these slow-perfusion conditions in the absence of inhibitors[52]. It is important to note that the variability is not caused by differences in sensor expression levels because the ratio signal is independent of the concentration of the sensor (S1 Fig).

**Fig 2:**
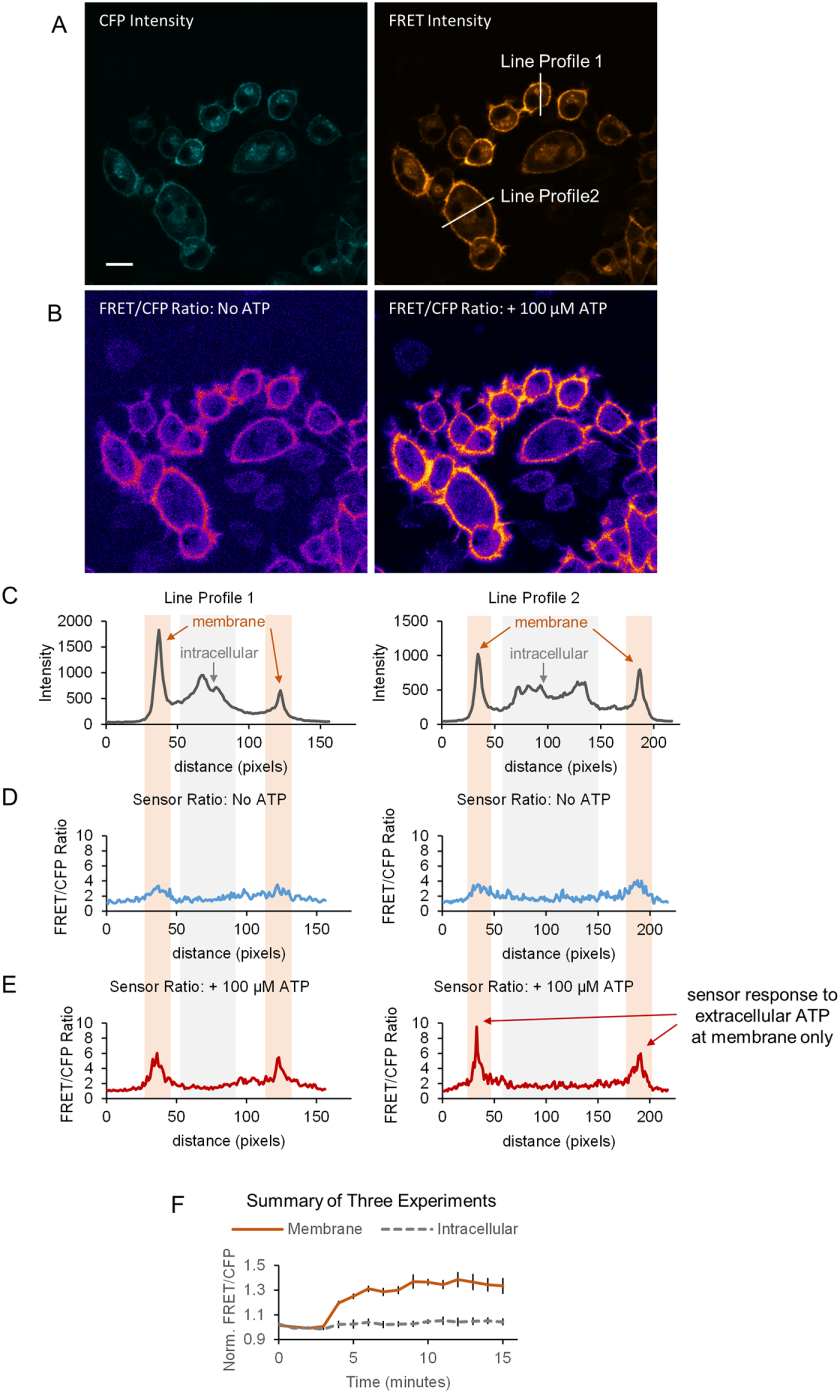
Surface-localized ecAT3.10 responds to the addition of extracellular ATP. High magnification confocal microscopy demonstrates that the ecAT3.10 ratio signal response to extracellular ATP occurs exclusively at the membrane. (A) Representative confocal fluorescence intensity images in the CFP and FRET channels show strong membrane localized signal and some intracellular puncta. Scale bar is 20 μm. (B) Pixel-by-pixel FRET/CFP ratio images before (left) and after addition of 100 μM ATP (right) illustrates a response at the membrane only. (C-E) Line profile analysis for the two example cells shown in (A). A 5-pixel width line region of interest was drawn through the membrane and peak areas of intracellular fluorescence. (C) The fluorescence intensity line profiles exhibit peaks at the membrane and also from intracellular locations that are likely ER/Golgi in origin. The line profiles from the ratio images shown in (B) before (D) and after (E) the addition of extracellular ATP clearly show that the ecAT3.10 ratio signal increases at the membrane and not from the intracellular sites. (F) After 3 minutes, 100 μM ATP was added. The time course shows that the membrane-localized ratio signals increase, but the signals from intracellular regions do not change (mean ± sem for three experiments with n = 32 cells, n = 60 cells, and n = 46 cells).

After demonstrating the reversibility of the sensor, we examined the spatial profile of the ecAT3.10 response in greater detail to show that ecAT3.10 is a faithful reporter of extracellular ATP. The sensor exhibited strong surface-targeted fluorescence signals, but intracellular punctate fluorescence was also apparent (Figs 1 and 2). Intracellular punctae have been observed during overexpression of fluorescent protein sensors by other groups, and it is attributed to excess protein resident in the endoplasmic reticulum[47]. Sensor responses were therefore analyzed for ratio signal changes within subcellular ROIs including the cell membrane and intracellular regions of interest using confocal microscopy. The confocal analysis confirmed that the fluorescence response originates exclusively from the membrane (Fig 2)). Furthermore, even in widefield imaging mode the whole-cell fluorescence signal response is reflective of the membrane response (S2 Fig). Thus, ecAT3.10 can be conveniently used with either confocal or widefield microscopy, and the subsequent experiments presented here utilized widefield microscopy.

**Fig 3:**
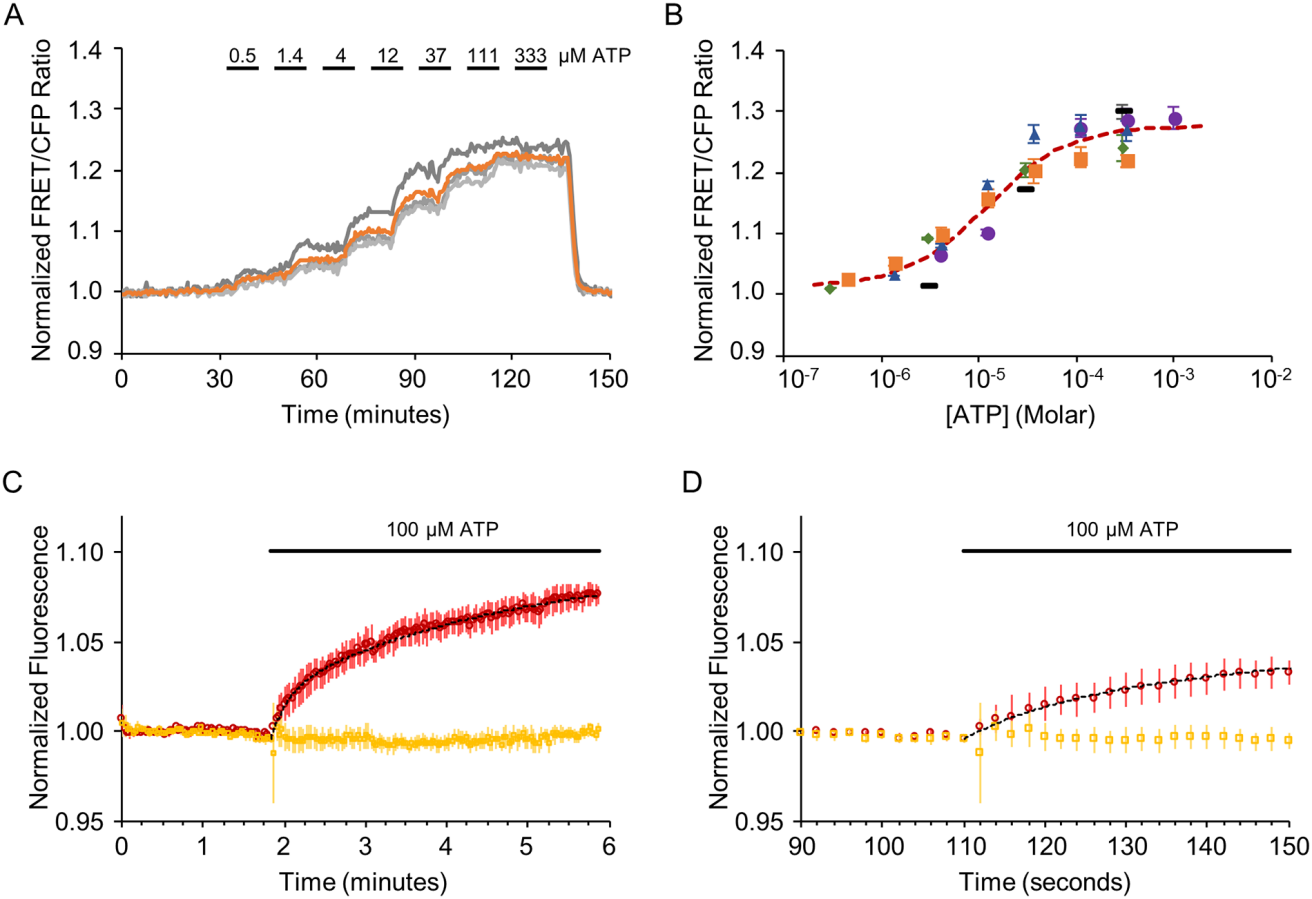
Characterization of ATP affinity and response kinetics. ecATeam3.10 exhibits a concentration-dependent change in FRET/CFP ratio signal. Live Neuro2A cells expressing ecAT3.10 were imaged at room temperature under continuous perfusion, during which increasing concentrations of extracellular ATP were washed in a stepwise fashion. (A) A representative time course from one experiment with four cells. Each grey trace represents the response from an individual cell in which the regions of interest were drawn around the membrane. The orange trace is the mean response. (B) Summary of ATP dose-response data from five independent experiments, each represented its own symbols. The orange squares represent data from (A), and the dashed red curve is fit to the mean of all experiments. In four of the five experiments, sufficient data was available for independent fitting to a Hill equation (mean ± sem, n = 4 experiments: K = 12 ± 5 μM, Ratio_max_/Ratio_min_ = 1.23 ± 0.01, n = 1.4 ± 0.2). A global fit of data from all five experiments was also performed for comparison. (fitting mean ± sem, n = 5 experiments: K = 11 ± 2 μM, Ratio_max_/Ratio_min_ = 1.28 ± 0.01, n = 0.9 ± 0.1). (C-D) ecAT3.10 responds to an increase in extracellular ATP within seconds. (C) Cells were imaged during fast perfusion wash in of 100 μM ATP, and the sensor exhibited an increase in FRET within seconds (n = 27 cells, 3 experiments; two-exponential fit: τ_fast_ = 13 ± 1 sec, τ_slow_ = 172 ± 18 sec). (D) An expanded view of the initial response shown in (C). Red, FRET fluorescence channel intensity. Yellow, YFP fluorescence channel intensity. Dotted black curve, two-exponential fit.

**Fig 4:**
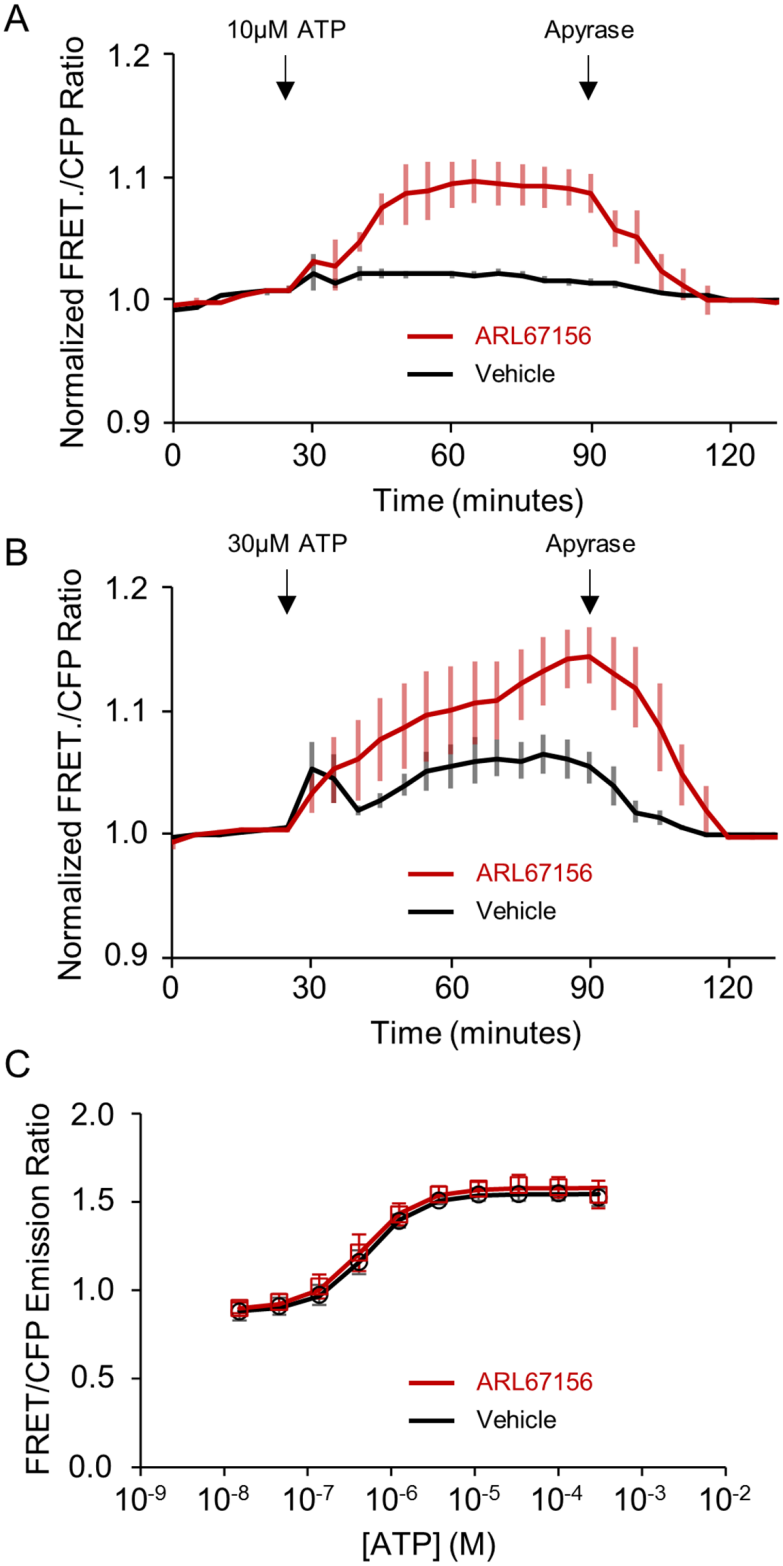
ecATeam3.10 detects ATP hydrolysis by ectonucleotidases. Live Neuro2A cells expressing ecAT3.10 were imaged under non-perfusion, static bath conditions. (A) The addition of 10 μM ATP did not elicit a response (black) unless the cells were pre-treated with the ectonucleotidase inhibitor ARL67156 at 100 μM (red). Apyrase addition degrades extracellular ATP confirming a reversible ATP-specific sensor response. (B) Ectonucleotidase inhibition by ARL67156 pre-treatment also potentiated responses when 30 μM ATP was added. Vehicle, black. ARL67156, red. (A) and (B), n = 6, 2 independent investigators. (C) ARL67156 does not directly affect the ATeam3.10 sensor response to ATP.

After our initial tests, we characterized the concentration range in which ecAT3.10 can detect extracellular ATP. Live Neuro2A cells expressing ecAT3.10 were imaged at room temperature with continuous bath perfusion to minimize cell-to-cell variability. Increasing concentrations of exogenous ATP from 0 μM to 1000 μM were washed onto the cells during real-time image acquisition, and steady-state was achieved before changing the extracellular ATP concentration (Fig 3). The sensor “on-cell” responses exhibited concentration-dependent increases of the ecAT3.10 ratio signal, and an apparent affinity equilibrium constant, K_app_ of 12 ± 5 μM (mean ± sem, n = 4 experiments), was determined when data were fitted with a Hill equation (Fig 3). Importantly, the apparent affinity of ATP for the purified soluble ATeam3.10 sensor was approximately 20-fold higher (K_app_ = 0.52 ± 0.02 μM, mean ± sem, n = 14 experiments) (Fig 4) compared to the “on-cell” response (Fig 3). Taken together, the difference between the K_app_ values for ATP between ecAT3.10 expressed on living cells and purified AT3.10 *in vitro* may reflect changes in the binding properties for ecAT3.10 imparted by surface-tethering of the sensor. There has been concern in the literature that glycosylation of ATeam can inactivate and alter the sensor’s properties[53, 54], but similar to other studies[55] we did not find evidence that glycosylation affects sensor function (S3 Fig). Despite the change in affinity, the sensitivity range overlapped with the reported physiological range of extracellular ATP from basal nanomolar to peak micromolar concentrations[56, 57].

We also characterized the kinetics with which the ecAT3.10 sensor responds to extracellular ATP. Neuro2A cells expressing ecAT3.10 were imaged at room temperature with continuous perfusion at a rate of 5 mL/min in a laminar flow fast perfusion chamber. In order to increase the acquisition speed, only the FRET and YFP fluorescence intensity channels were collected at 2 second intervals, which did not require any filter changes. The ecAT3.10 sensor responded within several seconds upon wash in of 100 μM ATP, and on average the response was best fit to a two exponential function with a fast time constant, τ = 13 ± 1 sec, and a slow time constant, τ = 172 ± 18 sec (n = 27 cells, 3 experiments) (Fig 3 and S4 Fig). Thus, the sensor can detect micromolar changes in extracellular ATP concentrations within seconds.

### ecAT3.10 reports extracellular ATP turnover by ectonucleotidase activity

We next demonstrated that ecAT3.10 can be used to detect the clearance of extracellular ATP via ectonucleotidase activity in cell cultures. During the measurement of the ATP affinity of ecAT3.10, continuous bath perfusion conditions were employed in order to continuously replenish ATP and control the extracellular ATP concentration. Physiologically, however, ectonucleotidases contribute to purinergic signaling dynamics by clearing extracellular ATP by hydrolysis[27, 58], thereby generating ADP, AMP, and adenosine, which can act independently as purinergic signals. In order to test ecAT3.10’s ability to detect endogenous ectonucleotidase activity[58], we performed experiments in which a single addition of exogenous ATP was made to cultured Neuro2A cells expressing ecAT3.10 in an imaging dish under static bath conditions without solution exchange. We initially used Neuro2A cells because they express the ectonucleotidase NTPDase1 (CD39)[51].

Our observations indicate that endogenous ectonucleotidases exhibit substantial activity to maintain low basal extracellular ATP levels around cultured cells. We first tested the addition of 10 μM ATP to ecAT3.10-expressing Neuro2A cells under static bath conditions. The concentration was chosen to mimic moderate to high physiological concentrations and because it demonstrated approximately half-maximal responses in the continuous perfusion experiments (Fig 3). Surprisingly, under static bath conditions no significant increase in the sensor ratio signal was detected (Fig 4), which is in stark contrast to bath perfusion experiments (Fig 3). However, pre-treatment with the ectonucleotidase inhibitor ARL67156[59] reconstituted the average peak response (vehicle, 1.02 ± 0.01 versus 100 μM ARL67156, 1.10 ± 0.02, mean ± sem, n = 6 experiments from 2 independent investigators, p = 0.009, t-test), and therefore the loss of signal change in the absence of ARL67156 is not caused by a loss of responsivity of the sensor itself. Potentiation of the response by ARL67156 indicates that high ectonucleotidase activity turns over extracellular ATP at a significant rate, at least at the cell surface. Importantly, 100 μM ARL67156 had no effect on the ability of purified soluble ATeam3.10 to detect ATP *in vitro*. The dose-response and K_app_ values for ATP in the presence and absence of ARL67156 were not significantly different (0.52 ± 0.02 μM, n = 14 versus 0.46 ± 0.07 μM, n = 5, p = 0.34, t-test) (Fig 4). Furthermore, the sensor ratio signal remained elevated until exogenous apyrase (3U/mL, high ATPase activity) was added to degrade the ATP (Fig 4). Apyrase itself does not affect the ability of ecAT3.10 to detect ATP (S5 Fig), and the decrease is not due to loss of sensor by internalization because the membrane-localized ratio signal is insensitive to sensor concentration. The apyrase addition caused a decrease in ecAT3.10 signal, demonstrating that the sensor remained responsive and was specific to ATP (Fig 4).

We next tested whether ectonucleotidase activity could clear surface ATP levels after the addition of a higher bulk concentration of extracellular ATP. Upon addition of 30 μM ATP, an initial increase in the sensor ratio signal was detected, and vehicle addition did not elicit a response (Fig 4 and S5 Fig). However, the sensor response to ATP was transient. After reaching a peak, within minutes the ecAT3.10 ratio signal spontaneously decreased, which is consistent with the hydrolytic breakdown of ATP by endogenous ectonucleotidase activity (Fig 4). Pretreatment with the ectonucleotidase inhibitor ARL67156 abrogated the spontaneous decrease and potentiated the peak response (vehicle, 1.07 ± 0.02 versus 100 μM ARL67156, 1.14 ± 0.02, mean ± sem, n = 6 experiments from 2 independent investigators, p = 0.02, t-test). The effect of ARL67156 supports the hypothesis that endogenous ectonucleotidases actively clear extracellular ATP, though hydrolytic activity was not able to completely suppress an increase in cell surface ATP when challenged with larger bulk ATP increases. In addition, the time-course of the response is complex under these static bath conditions because diffusion rate, clearance rate, and endogenous release (discussed below) rate all contribute to the overall dynamics. Overall, these data demonstrate that ecAT3.10 can be used to image and measure extracellular ATP clearance by endogenous ectonucleotidase activity on live cultured cells.

### ecAT3.10 detects nucleotide-stimulated endogenous ATP release

We next demonstrated that ecAT3.10 can be used to measure nucleotide-stimulated release of endogenous ATP from cells. It has previously been reported that P2X_7_ receptors can mediate ATP release from Neuro2A cells[60], and it is well established that ATP[61–65] and ADP[66, 67] can stimulate the release of ATP from a variety of cultured cells. The ATeam sensor itself can detect changes in ATP within seconds[43], which is well-matched to detect nucleotide-stimulated ATP release. Nucleotide-stimulated ATP release has previously been reported to cause an initial increase in extracellular ATP within seconds that can continue to increase and remain elevated over minutes[60–67], and in particular ATP-stimulated ATP release under static bath conditions has been observed to increase over tens of minutes[62].

**Fig 5:**
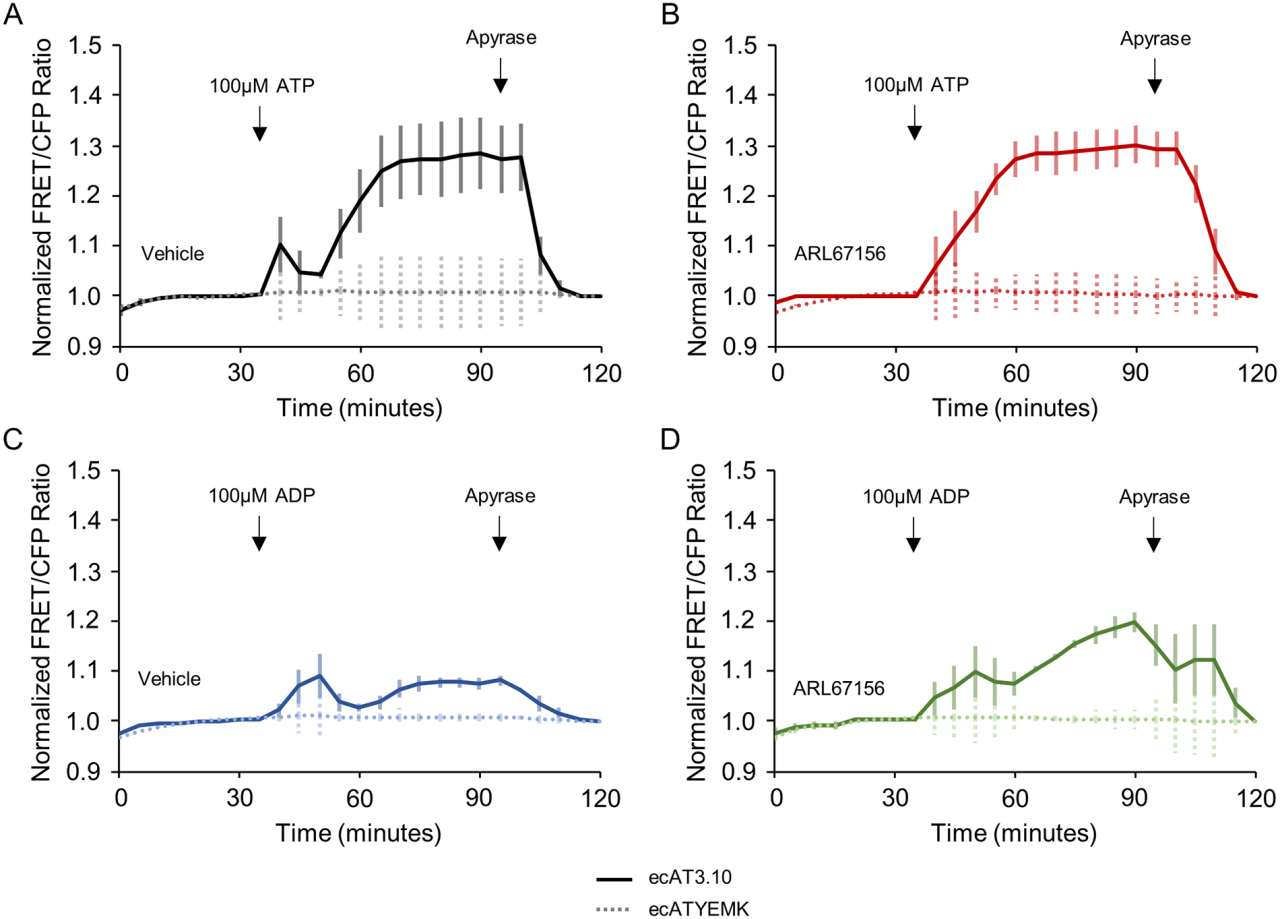
ecAT3.10 detects nucleotide-stimulated release of ATP from cells. (A) A biphasic response was apparent on average when 100 μM ATP was added at time = 30 minutes to the static bath of ecAT3.10-expressing Neuro2A cells. The decrease in signal at time = 45 minutes is due to ARL67156-sensitive ectonucleotidase activity, and the subsequent increase in signal after time = 55 minutes reports a secondary release of endogenous ATP from cells. (B) Pretreatment with 100 μM ARL67156 abrogates the transient decrease in ectonucleotidase activity. (C) Addition of 100 μM ADP also stimulate a release of ATP from cells, which was potentiated by pretreatment with ARL67156 (D). Apyrase addition degrades extracellular ATP confirming a reversible ATP-specific sensor response. Solid lines show average responses from ecAT3.10, and dashed traces show average responses from the negative control ecATYEMK sensor (n = 3).

Here, we imaged ecAT3.10-expressing Neuro2A cells under static bath conditions in order to observe persistent increases in extracellular ATP. In these experiments, technical replicates were imaged in a multi-well plate at 5 minute intervals. We observed a pronounced biphasic response following the addition of 100 μM ATP to ecAT3.10-expressing in Neuro2A cells (Fig 5) or HEK293 cells (S6 Fig). On average, a secondary increase in the ecAT3.10 ratio signal followed the initial decrease that we attribute to ARL67156-sensitive ectonucleotidase activity. This observation led to the hypothesis that the secondary increase is caused by a purinergic receptor-mediated release of intracellular ATP from cells. For example, such a release could plausibly be stimulated by ATP itself[60], one of its hydrolysis products, or possibly an intermediary purinergic ligand released by the initial ATP stimulation[68–73]. Therefore, we tested exogenous addition of ADP, adenosine, UDP, and UTP in comparison to ATP, each at a bulk extracellular concentration of 100 μM. The addition of ADP caused a robust increase in the ratio signal that was further potentiated by pretreatment with ARL67516 (Fig 5), whereas adenosine, UDP, and UTP did not elicit any response (S6 Fig). Clearly, there are complex differences between the ATP-elicited and ADP-elicited dynamics that likely reflect a number of signaling events that are well beyond the scope of this study. We therefore focued our study of the nature of the ADP-stimulated response to determine if the response is caused directly by ADP binding to the sensor or if ADP stimulates an endogenous release of intracellular ATP.

**Fig 6:**
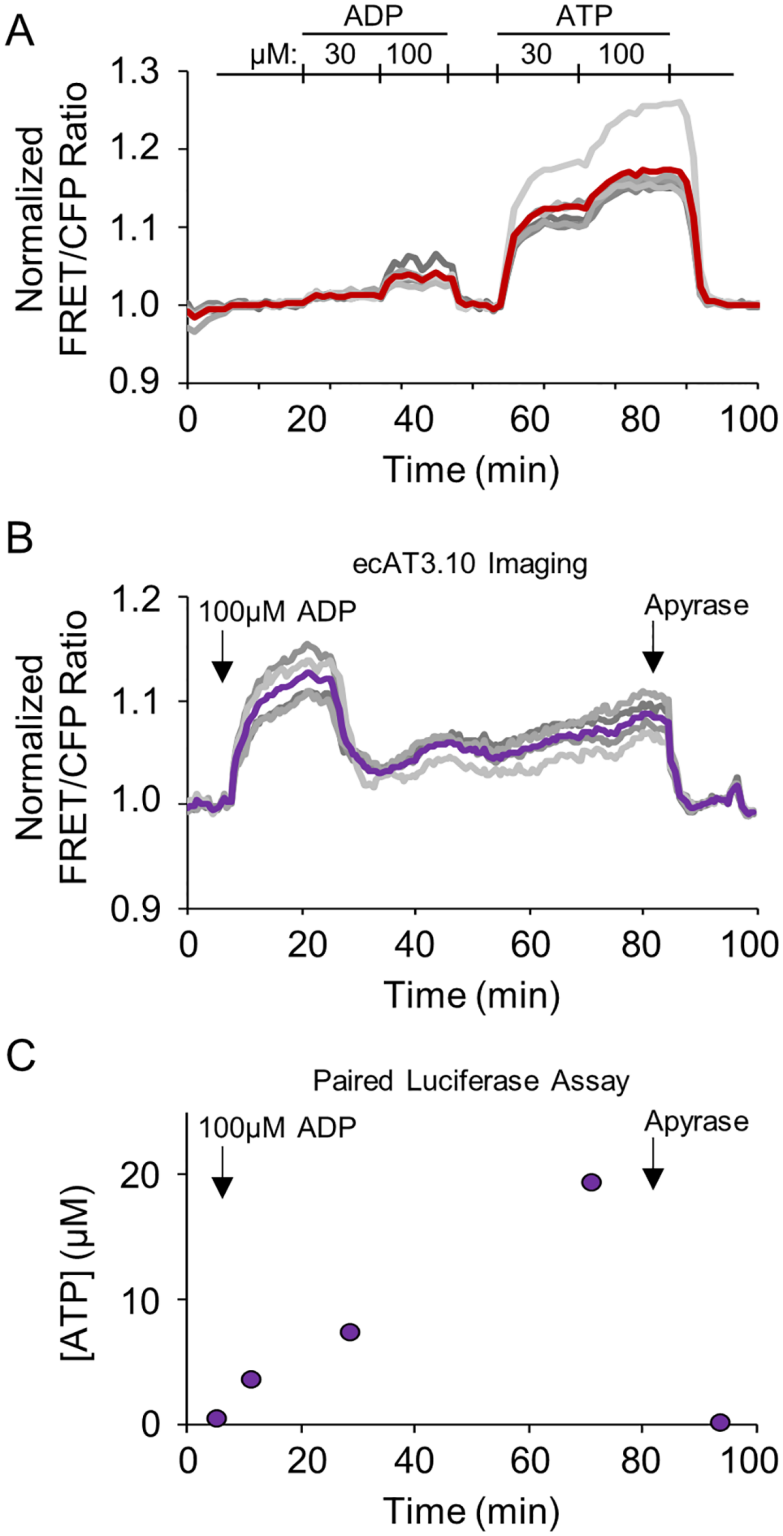
ecATeam3.10 detects ADP-stimulated release of endogenous ATP from live Neuro2A cells. (A) ecAT3.10-expressing Neuro2A cells were imaged under continuous perfusion conditions to demonstrate that ecAT3.10 has greater sensitivity to ATP compared to ADP. (B) Under static bath conditions, paired measurements by ecAT3.10 real-time imaging and endpoint luciferase assays demonstrate that ADP stimulates ATP release. In (A) and (B) grey traces are individual cells, and the bold trace is the cell mean. Arrows in (B) and (C) indicate the addition of ADP and apyrase, sequentially. In (C), time points represent samples from the bath solution taken during the ecAT3.10 imaging experiment shown in (B).

Our subsequent experiments indicate that the ecAT3.10 response is specific to ATP and not caused by an effect of ADP directly on the sensor. First, as a low affinity ligand for the soluble ATeam3.10 sensor, ADP could directly bind the sensor to produce an increase in the ratio signal. In fact, 100 μM ADP nearly saturates the purified soluble ATeam3.10 sensor, which suggests that ADP binding to ecAT3.10 could be responsible for the observed live-cell response from ecAT3.10-expressing Neuro2A cells[43]. However, the ATeam3.10 sensor is nearly 100-fold more selective for ATP compared to ADP (S7 Fig), and given our observation that the ATP response profile of surface-bound ecAT3.10 is drastically shifted relative to soluble ATeam3.10, we tested the response of ecAT3.10 to direct ADP binding with bath perfusion experiments (Fig 6). In contrast to the soluble sensor, ecAT3.10 showed a minimal fold increase in ratio signal upon wash-in of ADP (30 μM ADP, 1.009 ± 0.002; 100 μM ADP, 1.026 ± 1.005) compared to a subsequent wash-out of ADP and wash-in of ATP (30 μM ATP, 1.106 ± 0.009; 100 μM ATP, 1.14 ± 0.01, mean ± sem, n = 3 experiments). Although the ecAT3.10 is not perfectly specific for ATP compared to ADP, it may be improved with future engineering. However, the current sensor is 5–10 times more responsive to ATP, and thus, while a direct response to ADP binding may contribute a minor fraction of the signal, it does not account for the total magnitude of the response, supporting a stimulated release of ATP. Interestingly, these data are consistent with the approximate 20-fold affinity difference observed for ATP between ecAT3.10 and soluble ATeam3.10 (Fig 3). Second, we showed that the ADP-mediated increase in the sensor ratio signal was not due to a direct effect of the nucleotides on the individual fluorescent proteins in ecAT3.10. To test this we used a low affinity ATeam variant, ATeam1.03YEMK[43], to generate the surface-targeted sensor, ecATYEMK. In contrast to ecAT3.10, this negative control construct ecATYEMK showed no response to either ATP or ADP (Fig 5), confirming that the response is not a non-specific effect on the individual CFP or YFP. Taken together, these data show that the increase in ecAT3.10 ratio signal cannot be solely attributed to direct ADP binding to the sensor, and they suggest that the increase is cause by an ADP-stimulated release of cellular ATP.

To confirm that the ecAT3.10 response is measuring an ADP-stimulated release of ATP from the Neuro2A cells, we measured extracellular ATP levels with an orthogonal biochemical assay, the widely-accepted luciferase end-point assay. To carry out this experiment, cells were stimulated with 100 μM ADP and the real-time ecAT3.10 response was measured by fluorescence microscopy (Fig 6). Simultaneously during imaging, samples of the static bath solution were taken at intervals, and the extracellular ATP levels were determined with a commercial luciferase-based ATP detection kit (Fig 6). As expected, the addition of ADP caused an increase in the extracellular ATP concentration that, as measured by the luciferase detection method, grossly mirrored the temporal pattern of the ecAT3.10 signal. Specifically, both the real-time ecAT3.10 ratio signal and the end-point luciferase measurements increased following the addition of ADP, and both decreased following the addition of apyrase (n = 4 experiments) (Fig 6 and S7 Fig). We did observe differences in the detailed changes in extracellular concentration detected by ecAT3.10 versus the luciferase measurements, which reflect the differences between cell surface and bulk volume measurements[57, 74]. These observations confirm that ecAT3.10 can detect the ADP-stimulated release of endogenous ATP from Neuro2A cells.

**Fig 7:**
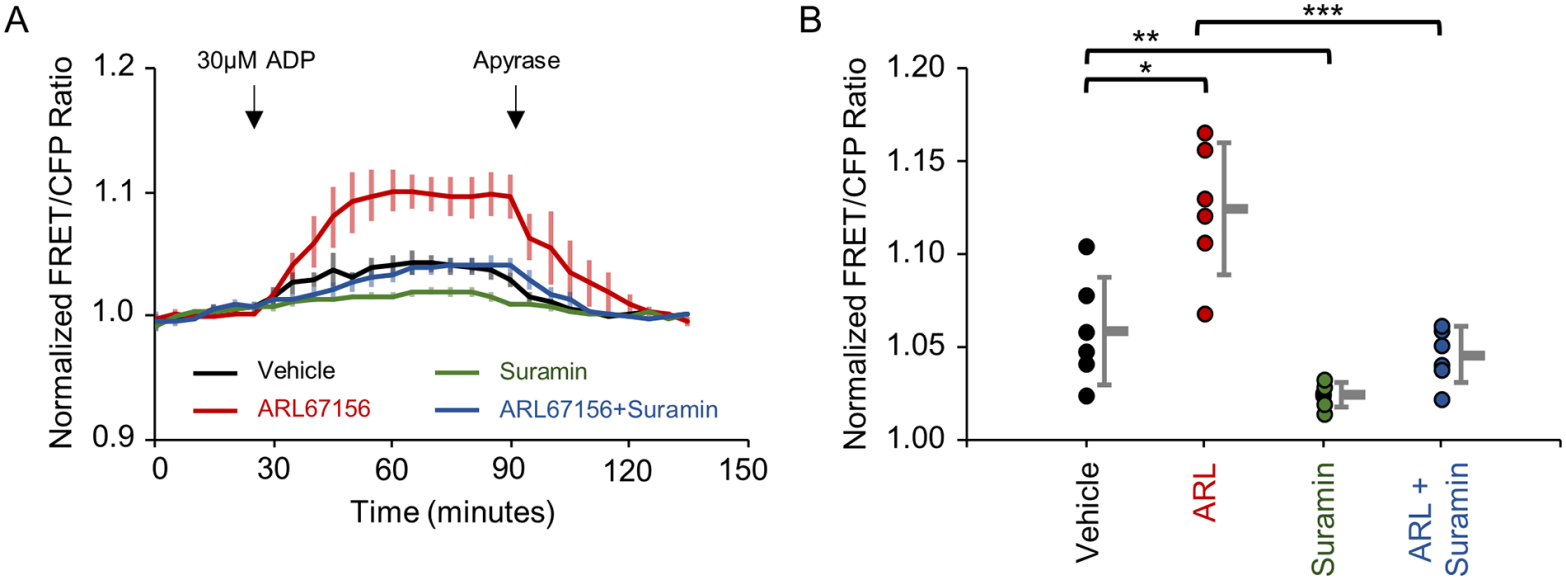
ADP-stimulated release of ATP release from Neuro2A cells may be mediated in part by purinergic pathways. (A) Average time course of ecAT3.10 ratio signal (baseline normalized, n = 6 experiments) from Neuro2A cells that were imaged under static bath conditions. 30 μM ADP was added to stimulate release of ATP. Pretreatment with 100 μM ARL67156 (red) potentiated the response in comparison to vehicle treatment (black). Treatment with 3.3 μM suramin (green) attenuated extracellular ATP levels compared to vehicle (black), and treatment with suramin in the presence of ARL67156 (blue) attenuated the release of ATP compared to ARL67156 alone (red). Apyrase addition degrades extracellular ATP confirming a reversible ATP-specific sensor response. (B) Summary of cell averaged peak responses for individual experiments. Peak responses: vehicle, 1.06 ± 0.01; ARL67156, 1.13 ± 0.01; suramin, 1.027 ± 0.002; ARL67156 plus suramin, 1.048 ± 0.006, mean ± sem. t-test, *p = 0.004, **p = 0.02, ***p = 0.0004. n = 6, 2 independent investigators.

ADP-stimulated accumulation of extracellular ATP may be mediated by purinergic receptor signaling pathways, as several P2Y receptor isoforms are known to be modulated by ADP[30]. Therefore, a non-specific purinergic receptor antagonist, suramin, was tested for its effects on the ADP-stimulated release of ATP from Neuro2A cells[3, 30, 32, 75]. Specifically, the ecAT3.10 response to ADP (30 μM) treatment was measured for Neuro2A cells that were pretreated with suramin (3.3 μM), either in the presence or absence of the ectonucleotidase inhibitor, ARL67156 (100 μM) (Fig 7). Consistent with ligand-stimulated ATP release from Neuro2A cells, pretreatment with ARL67156 potentiated the response to ADP which was reversed with subsequent apyrase treatment as expected (Fig 7). Furthermore, the ARL67156-potentiated ecAT3.10 response to ADP treatment was attenuated by suramin, suggesting that the ADP-stimulated release of ATP into the extracellular space was mediated by purinergic receptor signaling components (Fig 7). Suramin non-specifically targets P2X and P2Y receptors, therefore we tested the P2Y inhibitor, pyridoxalphosphate-6-azophenyl-2',4'-disulfonic acid (PPADS). Suramin and PPADS attenuated release; however, we discovered that PPADS directly altered the sensor response (S8 Fig). Despite this, the confluence of evidence described above indicates that treatment of Neuro2A cells with ADP ultimately caused an accumulation of extracellular ATP that was detected by ecAT3.10, providing a proof-of-concept validation that ecAT3.10 was able to detect cell-mediated extracellular ATP dynamics.

## Discussion

We have demonstrated that ecAT3.10 can be used to image and measure extracellular ATP dynamics in real-time, which is a significant advance because it provides a new tool to study purinergic signaling in live specimens. Extracellular ATP has been previously measured by a number of end-point biochemical and biophysical assays[3, 15, 21, 61–63, 76–78]. Real-time monitoring of purinergic signaling has also been achieved using ATP sniffer cells[79–81], enzyme-coupled optical fibers and electrodes[21, 25], and cell surface-tethered luciferase[74]. These methods have estimated that basal ATP levels in cell culture and in tissue are as low as nanomolar concentrations, whereas peak release levels have been estimated to reach micromolar concentrations, representing a concentration range spanning several orders of magnitude[3, 21, 63, 76–78]. Real-time measurements, including calibrated measurements from surface-tethered luciferase, are in agreement, though these measurements may have potentially underestimated surface ATP concentrations when not directly sampling at the cell surface[76, 78, 82, 83]. Surface-tethered luciferase could also underestimate surface levels because it requires access of a diffusible luciferin substrate and typically requires long integration times. To overcome this, a small organic fluorescent probe[84] has been used to monitor surface ATP levels in real-time on immune cells[76, 78], but it requires application of a lipidated probe. Overall, these methods variously suffer from limitations due to spatiotemporal resolution, specificity, exogenous substrate access, or sample destruction, which limit their use in live specimens[35]. A genetically-encoded sensor, such as ecAT3.10, offers the capability to quantitatively visualize purinergic signaling with minimal perturbation after gene transfer. To this end, we have demonstrated that ecAT3.10 is able to reversibly report changes in extracellular ATP in a physiologically relevant concentration range.

Physiologically, clearance of extracellular ATP is constitutive, and we demonstrated that ecAT3.10 can detect ARL67156-sensitive ectonucleotidase activity that serves to maintain a low surface ATP concentration on cultured cells. It is therefore important to take these biological activities into account when deciphering the sensor responses. We measured the concentration-response relationship between ATP and ecAT3.10 under continuous perfusion conditions to maximally hold the bulk ATP concentration constant. However, the cell surface is likely a privileged compartment[82, 83] where the observed ATP diffusion coefficient may be different from the bulk diffusion coefficient, and both biological clearance and release contribute to the net response[57, 63, 76–78]. For example, Neuro2A cells are known to express the ectonucleotidase CD39[51], but ecAT3.10 was able to reveal population heterogeneity at the level of single-cell analysis, which may be caused by cell-to-cell variation in CD39 activity that dampens the response magnitude. It is also possible that ectonucleotidase turnover of local surface ATP generates an ATP concentration gradient that increases into the bulk bath volume, which serves as a reservoir of diffusing ATP—though future experiments, beyond the scope of this study, are needed to test this compartmentation explicitly. Importantly, despite the variability in the response magnitude, the sensitivity of the sensor to ATP was consistent, supporting that ecAT3.10 reports physiological ATP dynamics. Overall, ecAT3.10 was able to detect clearance of extracellular ATP, suggesting that ecAT3.10 may prove useful in quantitatively characterizing CD39 activity, for example, that is linked to immunogenicity of various cancer cell types[10]. Furthermore, the high ectonucleotidase activity that we observed in cultured cells matches well with the physiological need to prevent non-specific activation of purinergic receptors.

To this end, we also provided evidence that ecAT3.10 could detect a purinergic receptor-mediated endogenous release of ATP as a proof-of-principle. It has been previously reported that Neuro2A cells express purinergic receptors[68–73] and can be stimulated to release ATP[60]. While a stimulated release event itself is expected to occur within seconds[20], it well established that extracellular ATP concentrations can remain elevated for several minutes, particularly under static bath conditions[60–67]. Similar to these previous reports, we observed that ecAT3.10 detected a suramin-sensitive increase in extracellular ATP following ADP treatment. Under these static bath conditions the increases occurred and persisted over several minutes, which is not attributed solely to the intrinsic kinetics of the sensor because the sensor itself can respond to changes in extracellular ATP levels within seconds. Instead, the persistant elevation in ATP likely reflect recurrent activation and release, which has been previously hypothesized[85]. Interestingly, despite its apparent affinity, K_app_ = 12 μM ATP, the sensor showed a strong response to stimulated release. Because the surface-tethered sensor does not require a diffusible substrate, the response correlates to the actual ATP dynamics at the cell surface. Hence, our observations suggests that surface ATP levels may indeed reach higher peak ATP concentrations than previously estimated. It will be interesting to use ecAT3.10 to study ATP release *in vivo* in the future because the extracellular volume constraints in intact tissue, in addition to differences between surface and bulk dynamics, are fundamental to the spatial and temporal components of purinergic signaling.

In summary, ecAT3.10 is a genetically-encoded and ratiometric fluorescent sensor of extracellular ATP that represents an important first-step in the creation of a new set of tools available for studying purinergic signaling in live specimens with single-cell resolution. Fluorescent protein-based sensors have emerged as powerful molecular tools to quantitatively visualize analyte dynamics in real-time, with subcellular resolution, and with the potential for cell-specific expression *in vitro* and *in vivo*. The ecAT3.10 is a CFP-YFP based sensor that is compatible with red fluorescent sensors, for example such as the calcium sensors[86–90] and pH sensors[91], that can be multiplexed to study signal transduction or improve quantitative calibration. Furthermore, the current work suggests that other available soluble ATP sensors can potentially be re-engineered as surface tethered sensors of extracellular ATP, though the current work also demonstrates the need for live-cell characterization. One of the potential challenges that calls for a broad toolset of sensors is the large concentration range of extracellular ATP in basal and stimulated conditions. For this reason, it will be important to engineer both ratiometric sensors[92] to study changes occurring over slower timescales as well as intensiometric sensors[93] to study faster events. By optimizing dynamic range, affinity ranges, and surface tether length, these sensors will ultimately enable purinergic researchers to study compartmentation of ATP dynamics *in vivo* in the future.

## Methods

### Materials

Standard chemicals and cell culture reagents were purchased from Sigma and ThermoFisher. ARL67156, suramin, and PPADS were purchased from Tocris.

### Data collection and analysis

All data collection and descriptions of replicates for data presented in each figure are discussed in S1 Table).

Unpaired, two-tailed Student’s t-test was used to test for differences in means. Mean values are reported with standard errors of the mean (mean ± sem).

### Sensor construction

#### ecAT3.10

The pCMV(MinDis).iGluSnFR plasmid (Addgene plasmid #41732), a modified version of the pDisplay vector that has the hemagglutinin (HA) tag removed and contains the iGluSnFR glutamate sensor, was used as a template for construction of the ecATeam sensors. The plasmid was restriction enzyme digested with BglII and SalI for removal of iGluSnFR sequence, but retention of the coding region for the IgK leader sequence and PDGFR transmembrane domain. The ATeam3.10 insert was prepared, as follows. The pRSetB-ATeam3.10 vector (from H. Imamura) was restriction enzyme digested with XbaI and EcoRI to divide the ATeam3.10 sequence into 2 fragments that separate the homologous cyan and yellow fluorescent protein sequences to allow for specific PCR amplification. Subsequently, the N-terminal ATeam3.10 fragment was amplified with the 5’-CAGGTTCCACTGGTGACAGAATGGTGAGCAAGGGCG–3’ and 5’–CACCATGAATTCCTTCATTTCGGCAACG–3’ oligonucleotide primer pair and the C-terminal fragment was amplified with the 5’–GAAATGAAGGAATTCATGGTGAGCAAGGGC–3’ and 5’–GATGAGTTTTTGTTCGTCGACCTTGTACAGCTCGTCCATGC–3’ oligonucleotide primer pair using Phusion polymerase (Fisher Scientific). Upon gel purification, the pCMV(MinDis) vector fragment and the two PCR-amplified ATeam3.10 fragments were used for a 3-fragment Gibson Assembly reaction (NEBuilder Gibson Assembly, New England Biolabs) that yielded the pCMV(MinDis)-ATeam3.10 construct (ecAT3.10).

#### ecAT1.03-YEMK

The pCMV(MinDis).iGluSnFR plasmid was also used as a template for construction of the ecAT1.03-YEMK construct. Here, 5’-CCGCCACAACATCGAGTACCTGCAGGTTGACGAACAAAAACTCATCTC-3’ and 5’-TTGCTCACCATACTCGAGTAGTCACCAGTGGAACCTG-3’ oligonucleotide primers were used with Phusion polymerase to PCR amplify a fragment of the pCMV(MinDis).iGluSnFR plasmid that retains the IgK leader sequence and PDGFR transmembrane domain, but is devoid of the iGluSnFR sequence. The insert fragment containing AT1.03-YEMK was prepared by restriction enzyme digest of pcDNA-AT1.03(YEMK) (from H. Imamura) with XbaI and HindIII. The final pCMV(MinDis)-AT1.03-YEMK (ecAT1.03-YEMK) construct was generated by Gibson Assembly (NEBuilder, New England Biolabs) of the insert fragment with the PCR amplified pCMV(MinDis) vector fragment.

Upon preparation of plasmid DNA, all coding sequences of the final ecATeam constructs were confirmed by Sanger sequencing at Genewiz (South Plainfield, NJ).

### Cell line maintenance and transfection

N2A cells (ATCC CCL-131), HEK293 cells (ATCC CRL-1573), and SK-MEL-5 (ATCC HTB-70) were cultured in Dulbecco’s modified Eagle’s medium containing 4.5 g/L glucose, 2 mM glutamine, and supplemented with 10% cosmic calf serum or 10% fetal bovine serum and were maintained at 37°C and 5% CO_2_ in a humidified incubator. N2A cells were confirmed to be mycoplasma free as assessed by the Universal Mycoplasma Testing Kit (ATCC). For imaging experiments, cells were seeded, as indicated, onto nitric acid-cleaned and washed 18-mm #1.5 coverslips, or into Cellvis 12-well or 24-well glass bottom plates with high performance #1.5 cover glass that were cleaned with NaOH/H_2_O_2_, washed, and pre-coated with poly-D-lysine. Cells were then transfected with plasmid DNA for expression of ecATeam sensors by living cells using the Effectene (Qiagen) transfection reagent as described in the manufacturer’s protocol.

### Live-cell fluorescence microscopy and image analysis

Cells were prepared for microscopy by exchanging cell growth media for imaging solution consisting of 15 mM HEPES, 1.25 mM NaH_2_PO_4_, 10 mM glucose, 120 mM NaCl, 3 mM KCl, 2 mM CaCl_2_, 1 mM MgCl_2_, and 3 mM NaHCO_3_ (pH 7.3) and cells were equilibrated at room temperature for at least 20 min prior to imaging. All microscopy experiments were performed at ambient room temperature, either during continuous perfusion (~1.5 mL/min flow rate) of imaging solution (or indicated treatment), or in the presence of a static bath of imaging solution where treatments were added by pipette to the bulk solution of the static bath. Cells were imaged using an Olympus IX83 fluorescence microscope with a 20X/0.75 NA objective illuminated by a Lumencor SpectraX light engine and equipped with an Andor Zyla 4.2 sCMOS camera. The ecATeam sensor activity was measured by examining the fluorescence intensities in CFP, CFP-YFP FRET, and YFP channels. Specifically, the ATeams were excited in the CFP and CFP-YFP FRET channels using a 438/29 nm bandpass filter, and emission was collected through 470/24 and 540/30 nm bandpass filters for the CFP and CFP-YFP FRET channels, respectively. The fluorescence in the YFP channel was excited using a 510/10 nm bandpass filter and emission was collected through a 540/30 nm bandpass filter. Excitation light from all fluorescence channel measurements was blocked by the ET-ECFP/EYFP/mCherry multiband beamsplitter (Chroma 69008bs). The microscope components and image acquisition were controlled by the Andor iQ3 software and the ImageJ/FIJI software package was used to analyze all images for each experiment, as specified. For fluorescence images, the mean and standard deviation of background intensities were measured for each fluorescence channel in every field of view. For each fluorescence channel, the mean background intensity was subtracted from every image of the associated imaging set. Background masks were then created with minimum thresholds of two times the mean background intensity. The background masks were applied to the fluorescence intensity images and fluorescence ratio images were created by pixel-by-pixel division of the background masked individual fluorescence channels. Regions of interest that encompassed whole cells or cell membranes, as indicated, were drawn and the mean fluorescence ratios of pixels within each region of interest were calculated.

For kinetic measurements, experiments were performed at ambient room temperature using a diamond-shaped laminar flow fast perfusion chamber (Warner RC-25F). Imaging solution was perfused at a rate of 5 mL/min, and inhibitors were not used. Images were collected at 2 second intervals for the FRET and YFP fluorescence emission channels. Data was fit to a two-exponential association function,y = y_0_ + A_1_·(1-e^(-t/τ1)^) + A_2_·(1-e^(-t/τ2)^).

Confocal microscopy was carried out on a Nikon A1 confocal system with a Ti-E microscope base, using a 60X/1.49 NA Nikon apo TIRF objective at room temperature. The 457 nm line of a mult-argon laser was used for excitation, and CFP and FRET channel fluorescence intensities were collected using 482/35 nm and 525/50 nm bandpass filters.

### Luciferase assay

Neuro2A cells were maintained, seeded onto nitric acid-cleaned 18-mm #1.5 glass coverslips, transfected, and prepared as described for live-cell fluorescence microscopy experiments in imaging solution. Cells were imaged for ecAT3.10 fluorescence in a coverslip chamber under static bath conditions, without solution exchange. While imaging ecAT3.10 in Neuro2A cells, 25 μl of extracellular imaging solution was removed at various time points and immediately frozen and stored at -80°C until assay. The ATP concentrations in the extracellular imaging solution aliquots were measured using a commercial luminescence ATP detection assay system (ATPlite, PerkinElmer) and quantified compared to an ATP standard curve.

### ATeam3.10 expression and purification

*E*. *coli* strain BL-21 expressing pRSetB-ATeam3.10 was cultured in Auto Induction Media (AIM) at 37°C for 12h then at room temp for 36h. Cells were then pelleted, resuspended in lysis buffer (25 mM Sodium Phosphate Buffer [pH 7.8], 500 mM NaCl, 10 mM Imidazole, 5% Glycerol, 0.1% Triton X-100, 1mM PMSF, 1mM DTT, and 0.2 mg/mL Lysozyme), and lysed by sonication. Lysate was then centrifuged at 30,000xg for 30min at 4°C, and passed through a 0.45 μm syringe. The sample was then run over a Ni column (GE Chelating Sepharose column charged with Ni2+). The column was then washed with wash buffer A (25 mM Sodium Phosphate Buffer [pH 7.8], 500 mM NaCl, 20 mM Imidazole, 5% Glycerol), wash buffer B (25 mM Sodium Phosphate Buffer [pH 7.8], 500 mM NaCl, 40 mM Imidazole, 5% Glycerol), then elution buffer (25 mM Sodium Phosphate Buffer [pH 7.8], 500 mM NaCl, 150 mM Imidazole, 5% Glycerol). The fractions containing purified ATeam 3.10 were dialyzed in storage buffer (5 mM MOPS Buffer [pH 7.3], 300 mM NaCl, 10% Glycerol), with two changes at 4°C and stored at -80°C for future use.

### Purified AT3.10 in vitro spectroscopy

Protein concentration was determined using a standard procedure micro Bradford Assay (Thermo Scientific). The protein was diluted to 0.125 μM in assay buffer (50 mM MOPS-KOH, pH 7.3, 50 mM KCl, 0.5 mM MgCl2, and 0.05% Triton X-100) and fluorescence was measured using a BioTek Synergy H4 microplate reader at room temperature, in the absence and presence of ATP, ADP, ARL67156, PPADS, or Suramin. For all filter based measurements, a 420/50 nm excitation and 485/20 and 528/20 nm emission filters were used.

## Supporting information

S1 Table Description of replicates for data presented in each figure. (PDF)

S2 Table Fig 3B [ATP] dose-response data and fitting. (PDF)

S3 Table Purified protein dose-response data and fitting. (PDF)

S4 Table Summary of peak responses shown in Fig 7B. (PDF)

S1 Fig Additional characterization of ecAT3.10 demonstrating that sensor expression does not buffer ATP and that the sensor faithfully reports changes in extracellular ATP. The ecAT3.10 ratio signal is independent of sensor concentation and expression level. (A-C) Widefiedl fluorescence microscopy examples of single-cell responses that were averaged in Fig 1 (cells 3, 14, 34). Changes in the FRET, YFP, and CFP fluorescence intensity channels are shown. The FRET/CFP ratio signal is independent of expression level, reflected by mean YFP fluorescence intensity, and the peak (D) whole-cell and (E) membrane responses are independent of expression level.
(PDF)

S2 Fig Using widefield microscopy, the spatial localization of the ratio signal change was analyzed for a subset of cells shown in Fig 1, confirming that the ecAT3.10 signal change primarily occurred from the perimeter (red, top trace) and not the interior (green, bottom trace). The whole cell average (black, middle trace) is reflective of the surface ecAT3.10 signal (n = 10 cells). Values and solid line traces are cell means, and errors and error bars are standard errors of the means.
(PDF)

S3 Fig The ecAT3.10 sensor is not affected by glycosylation. (A) Gel-shift assay in which gel fluorescence is imaged first followed by Coomassie staining of the same gel to visualize the fetuin controls. Treatment of cell lysates (lanes 4–5) or live cells (lanes 6–7) for 1 hour at 37°C with PNGaseF and O-glycosidase (from NEB in PBS supplemented with 1 mM CaCl_2_, 1 mM MgCl_2_, and 10 mM HEPES, pH 7.4; Hall *et al*. 2014 FEBS Open Bio. 4:892; Dellisanti *et al*. 2007 Nat. Neurosci. 10:953) does not cause a gel shift in the ecAT3.10 fluorescence band relative to untreated cells, indicating that the sensor is not glycosylated. Coomassie staining confirms that enzyme activity causes a gel shift for the non-fluorescent control protein fetuin (lanes 8–9). Samples in lanes 2–7 were left unboiled to preserve sensor fluorescence, and fluorescence was visualized with shortwave UV excitation. (C) Pre-treatment of cells for 1 hour at 37°C with PNGaseF, O-glycosidase, and neuramidase does not change the live-cell ATP dose response of ecAT3.10.
(PDF)

S4 Fig Additional characterization of the response kinetics. (A) The ecAT3.10 sensor responds within seconds to ATP addition. Individual cell responses (left panels) and mean (± standard error of the mean) response (right panels) for each of three experiments that are averaged in Fig 2. n = 9 cells per experiment.
(PDF)

S5 Fig Ectonucleotidase inhibitor ARL67156 (100 μM) potentiates the ecAT3.10 ratio signal in response to 30μM ATP addition under static bath conditions in Neuro2A cells, but the (A) vehicle and (B) ARL67156 alone do not elicit a response. (C) Apyrase does not prevent ecAT3.10 response to extracellular ATP. 5 units/mL apyrase (high ATPase activity) was washed in at t = 37 minutes, and no change was observed. An addition of 100 μM ATP and 4.3 units/mL apyrase at t = 47 min also did not elicit a response. Cells were superfused with imaging solution to wash out apyrase and ATP at t = 62 min. At t = 77 min an addition of 100 μM ATP caused an increase in the ecAT3.10 ratio signal that could be reversed upon washout at t = 90 min, confirming function of the sensor following apyrase treatment.
(PDF)

S6 Fig (A) ATP and ADP stimulate ATP release in ecAT3.10-expressing Neuro2A and HEK293 cells, but adenosine does not. (B) UTP, and UDP do not stimulate ATP release in Neuro2A cells.
(PDF)

S7 Fig (A) Paired imaging and luciferase experiments. Top panels show ecAT3.10 imaging experiments with Neuro2A cells stimulated to release ATP by 100μM ADP, which was subsequently degraded by apyrase addition. Bottom panels show paired luciferase ATP end-point assays in which time points represent samples take from the bath from paired imaging experiments in the top panels. (B) In vitro dose-response of purified AT3.10 for ATP (K_app_ = 0.52 ± 0.02 μM, mean ± sem, n = 14 experiments) and ADP (K_app_ = 46 ± 5 μM, mean ± sem, n = 9 experiments) demonstrates a nearly 100-fold selectivity for ATP over ADP in solution.
(PDF)

S8 Fig High micromolar concentrations of suramin and PPADS directly affect the ATP concentration response of purified soluble ATeam3.10 at room temperature. (Left) The presence of 100 μM ARL67156 or 3.3 μM suramin does not affect the sensor. (Right) At concentrations >10μM both suramin and PPADS significantly alter the sensor characteristics.
(PDF)
