# Supplementary material for: Imaging extracellular ATP with a genetically-encoded, ratiometric fluorescent sensor
Source: PLoS One. 2017 Nov 9;12(11):e0187481. doi: 10.1371/journal.pone.0187481 (PMC5679667; doi:10.1371/journal.pone.0187481)
Supplement: S1 Table — (PDF) [file pone.0187481.s001.pdf]

Table S1. Discussion of replicates.

<table>
<tr><th>Figure</th><th># Investigators</th><th>n # replicates</th></tr>
<tr><td>Figure 1</td><td>1 - J.M.C.</td><td>n=1</td></tr>
<tr><td colspan="3">J.M.C. collected the data, and J.M.C. and M.T. analyzed the data.

A single replicate is shown in Figure 1 because it is presented as a basic demonstration of the ecAT3.10 sensor response. 41 cells in total were analyzed and presented in Figure 1B-D. For Figure 1E, a subset of 10 cells, shown in Figure 1F, were analyzed.</td></tr>
<tr><td>Figure 2</td><td>1 – J.M.C.</td><td>n=3</td></tr>
<tr><td colspan="3">J.M.C. collected, and J.M.C. and M.T. analyzed the data.

Figure 2 is a representative replicate. Figure 2F summarizes data from 3 replicates in which 32 cells, 60 cells, and 46 cells were analyzed.</td></tr>
<tr><td>Figure 3</td><td>1 - J.M.C</td><td>n=5</td></tr>
<tr><td colspan="3">J.M.C. collected and analyzed the data.

Figure 2A is a representative replicate with 3 cells. Figure 2B summarizes data from 5 replicates in which 6 cells, 7 cells, 14 cells, 9 cells, and 3 cells, respectively were analyzed.</td></tr>
<tr><td>Figure 4</td><td>3 - J.M.C., S.R., S.A.V.</td><td>var</td></tr>
<tr><td colspan="3">J.M.C., S.R., and S.A.V. collected the data. J.M.C., S.R., S.A.V., and M.T. analyzed the data.

In Figure 3A, 4-5 cells were analyzed per replicate, 6 replicates were carried out, and a total of 25 cells were analyzed for the vehicle condition. For the ARL67156 condition, 4-5 cells were analyzed per replicate, 6 replicates carried out, and a total of 25 cells were analyzed.

In Figure 3B, 4 cells were analyzed per replicate, 6 replicates were carried out, and 24 cells total were analyzed for vehicle. For ARL67156, 4 cells were analyzed per replicate, 6 replicates were carried out, and a total of 24 cells were analyzed.

Figure 3C, technical duplicates were performed for all protein assays. For the vehicle condition, data from 14 replicates were collected, and for the ARL67156 condition, data from 5 replicates were collected.</td></tr>
<tr><td>Figure 5</td><td>2 - S.R., S.A.V.</td><td>n=3</td></tr>
<tr><td colspan="3">S.R. and S.A.V. collected the data. S.R., S.A.V., and M.T. analyzed the data.

In the vehicle condition in Figure 4A, data was collected from 3 replicates for the ecAT.310 (42 cells, 32 cells, 33 cells) and 3 replicates for the ecATYEMK negative control (34 cells, 29 cells, 50 cells).

In the ARL67157 condition in Figure 4B, data was collected from 3 replicates for the ecAT3.10-expressing cells (15 cells, 9 cells, 32 cells) and 3 replicates for the ecATYEMK negative control (15 cells, 32 cells, 29 cells).

In the vehicle condition in Figure 4C, data was collected from 3 replicates for the ecAT3.10-expressing cells (23 cells, 29 cells, 36 cells) and 3 replicates for the ecATYEMK negative control (23 cells, 27 cells, 39 cells).

In the ARL67157 condition in Figure 4D, data was collected from 3 replicates for the ecAT3.10-expressing cells (21 cells, 12 cells, 24 cells) and 3 replicates for the ecATYEMK negative control (21 cells, 30 cells, 39 cells).</td></tr>
<tr><td>Figure 6 + SI Fig. 7</td><td>1 - J.M.C</td><td>n=4</td></tr>
<tr><td colspan="3">J.M.C. collected the data. J.M.C. and M.T. analyzed the data.

In Figure 5A one replicate is shown, and data from 3 replicates were collected (6 cells, 6 cells, 6 cells).

In Figure 5B-C one replicate is shown for the paired imaging and luciferase assays. Data from 4 replicates were collected and 4 cells per replicate were analyzed from the paired imaging data.</td></tr>
<tr><td>Figure 7 + SI Fig. 8</td><td>2 - J.M.C., S.R., S.A.V.</td><td>n=6</td></tr>
<tr><td colspan="3">J.M.C., S.R. and S.A.V. collected the data. J.M.C., S.R., S.A.V., and M.T. analyzed the data.

In Figure 6A, data from 6 replicates were collected for each condition, with 4-5 cells analyzed per replicate. Unpaired, two-sided Student's t-test was used for comparisons.

Supplemental Figure 6: Technical duplicates for all replicates. Vehicle and ARL67156 curves are from Figure 3. 3.3μM suramin, 3 replicates; 10μM suramin 3 replicates, 30μM suramin 7 replicates, 30μM PPADS 4 replicates, 30μM suramin + 30μM PPADS 5 replicates</td></tr>
</table>
