# Supplementary material for: Imaging extracellular ATP with a genetically-encoded, ratiometric fluorescent sensor
Source: PLoS One. 2017 Nov 9;12(11):e0187481. doi: 10.1371/journal.pone.0187481 (PMC5679667; doi:10.1371/journal.pone.0187481)
Supplement: S2 Table — (PDF) [file pone.0187481.s002.pdf]

Table S2. Figure 2B [ATP] dose-response data and fitting.

Replicate 1		Replicate 2		Replicate 3		Replicate 4		Replicate 5	
[ATP] (μM)	F/F_{base}	[ATP] (μM)	F/F_{base}	[ATP] (μM)	F/F_{base}	[ATP] (μM)	F/F_{base}	[ATP] (μM)	F/F_{base}
0	1.001±0.001	0	0.999±0.001	0	0.999±0.001	0	1.000±0.001	0	1.001±0.001
3	1.012±0.003	4	1.067±0.004	1.37	1.032±0.003	0.3	1.010±0.002	0.457	1.025±0.005
30	1.172±0.012	12	1.102±0.003	4.11	1.081±0.008	3	1.09±0.01	1.37	1.05±0.01
300	1.30±0.02	37	1.21±0.02	12.3	1.18±0.02	30	1.20±0.02	4.11	1.10±0.02
		111	1.27±0.02	37	1.26±0.02	300	1.24±0.03	12.3	1.16±0.02
		333	1.29±0.02	111	1.28±0.02			37	1.20±0.02
		1000	1.29±0.02	333	1.27±0.02			111	1.23±0.01
								333	1.22±0.01
Hill Fit Parameters									
Min	n.d.		1.054±0.004		1.024±0.003		0.995±0.002		1.016±0.004
Max	n.d.		1.29±0.02		1.28±0.02		1.25±0.03		1.228±0.009
K (μM)	n.d.		26±1		10. ±1		5.6±0.09		7±1
n	n.d.		1.7±0.1		1.80±0.08		0.96±0.04		1.10±0.04

*F/F_{base} is the fold-change over baseline, mean±sem.
**n.d. not determined
*** Fitted parameters, mean±fitting error.
