# Supplementary material for: Imaging extracellular ATP with a genetically-encoded, ratiometric fluorescent sensor
Source: PLoS One. 2017 Nov 9;12(11):e0187481. doi: 10.1371/journal.pone.0187481 (PMC5679667; doi:10.1371/journal.pone.0187481)
Supplement: S3 Table — (PDF) [file pone.0187481.s003.pdf]

Table S3. Purified protein dose-response data and fitting.

[ATP] (µM)	Vehicle	100µM ARL67156	3.3µM suramin	10µM suramin	30µM suramin	30µM PPADS	30µM suramin +30µMPPADS
0	0.86±0.01	0.89±0.01	0.813±0.002	0.863±0.003	0.94±0.02	0.562±0.006	0.78±0.03
0.02	0.88±0.01	0.90±0.01	0.828±0.003	0.863±0.008	0.94±0.02	0.55±0.01	0.77±0.03
0.05	0.91±0.01	0.93±0.02	0.847±0.002	0.877±0.005	0.93±0.02	0.55±0.01	0.76±0.03
0.14	0.97±0.02	1.02±0.03	0.917±0.006	0.912±0.004	0.94±0.02	0.563±0.005	0.77±0.03
0.41	1.16±0.02	1.21±0.05	1.091±0.003	1.018±0.003	0.97±0.02	0.61±0.02	0.78±0.03
1.2	1.39±0.01	1.43±0.03	1.37±0.02	1.252±0.007	1.02±0.02	0.71±0.03	0.84±0.04
3.7	1.508±0.006	1.54±0.02	1.49±0.01	1.44±0.02	1.12±0.03	0.83±0.03	0.95±0.04
11	1.54±0.01	1.57±0.02	1.53±0.01	1.50±0.02	1.33±0.02	0.91±0.03	1.06±0.04
33	1.55±00.1	1.60±0.03	1.52±0.02	1.52±0.02	1.39±0.02	0.92±0.04	1.10±0.04
100	1.55±0.01	1.58±0.03	1.547±0.008	1.52±0.02	1.41±0.01	0.94±0.03	1.11±0.04
300	1.52±0.01	1.54±0.03	1.539±0.008	1.52±0.01	1.419±0.009	0.94±0.03	1.11±0.05
Hill Fit Parameters							
Min	0.88±0.01	0.892±0.006	0.83±0.03	0.866±0.005	0.92±0.02	0.548±0.08	0.76±0.03
Max	1.54±0.01	1.58±0.03	1.538±0.006	1.52±0.01	1.43±0.01	0.94±0.03	1.12±0.05
K (µM)	0.52±0.02	0.46±0.07	0.576±0.008	0.96±0.02	3.5±0.04	1.8±0.3	4±1
n	1.44±0.06	1.4±0.1	1.45±0.05	1.403±0.008	1.33±0.07	1.4±0.2	1.5±0.1

*Values are raw CFP/FRET ratios, mean±sem.
** Fitted parameters, mean±fitting error.
