# Supplementary material for: Imaging extracellular ATP with a genetically-encoded, ratiometric fluorescent sensor
Source: PLoS One. 2017 Nov 9;12(11):e0187481. doi: 10.1371/journal.pone.0187481 (PMC5679667; doi:10.1371/journal.pone.0187481)
Supplement: S4 Table — (PDF) [file pone.0187481.s004.pdf]

Table S4. Summary of peak responses shown in Fig 6B.

	Vehicle	ARL67156	Suramin	ARL+Suramin
Replicate 1	1.058±0.002	1.16±0.03	1.026±0.003	1.042±0.003
Replicate 2	1.042±0.004	1.12±0.02	1.018±0.003	1.038±0.001
Replicate 3	1.025±0.002	1.111±0.006	1.023±0.004	1.053±0.008
Replicate 4	1.05±0.01	1.07±0.01	1.026±0.003	1.027±0.006
Replicate 5	1.08±0.01	1.13±0.01	1.033±0.006	1.060±0.008
Replicate 6	1.10±0.01	1.17±0.05	1.033±0.004	1.07±0.01

*Peak fold-change over baseline, mean±sem.
