# Supplementary figures and images for: Imaging extracellular ATP with a genetically-encoded, ratiometric fluorescent sensor
Source: PLoS One. 2017 Nov 9;12(11):e0187481. doi: 10.1371/journal.pone.0187481 (PMC5679667; doi:10.1371/journal.pone.0187481)

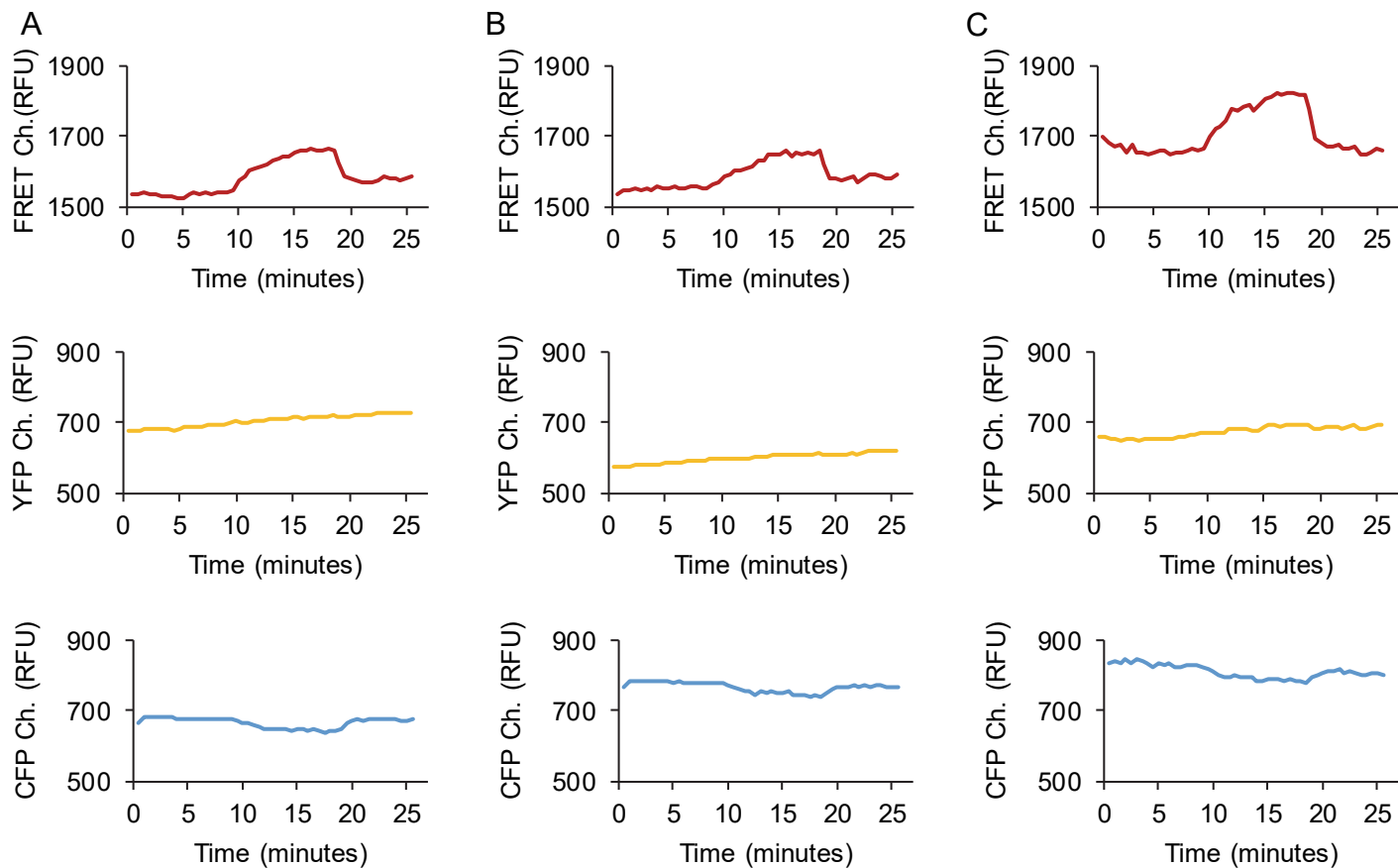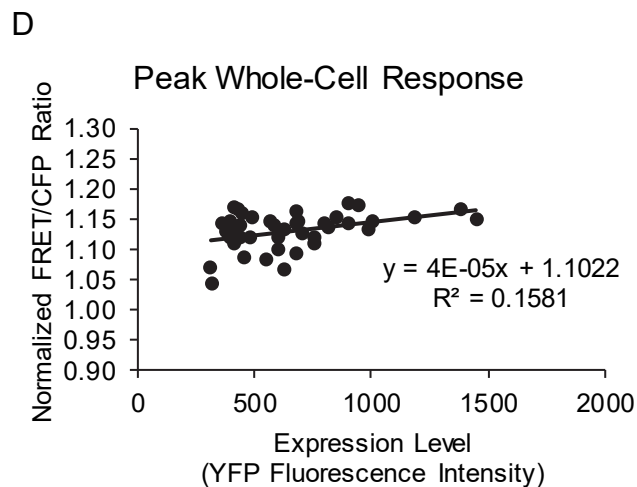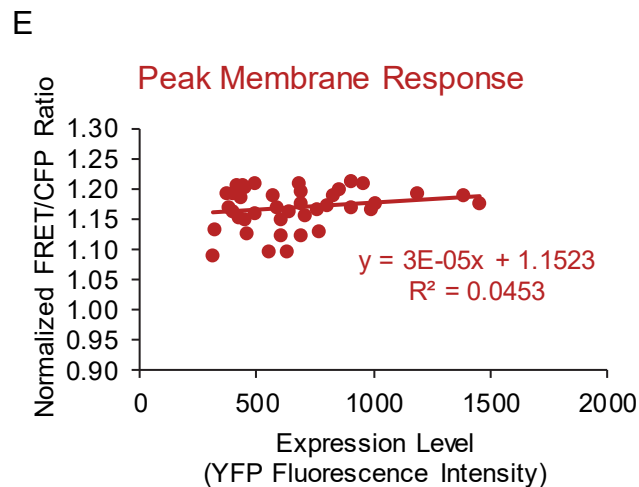

Supplement: S1 Fig — The ecAT3.10 ratio signal is independent of sensor concentation and expression level. (A-C) Widefiedl fluorescence microscopy examples of single-cell responses that were averaged in Fig 1 (cells 3, 14, 34). Changes in the FRET, YFP, and CFP fluorescence intensity channels are shown. The FRET/CFP ratio signal is independent of expression level, reflected by mean YFP fluorescence intensity, and the peak (D) whole-cell and (E) membrane responses are independent of expression level. (PDF) [file pone.0187481.s005.pdf]

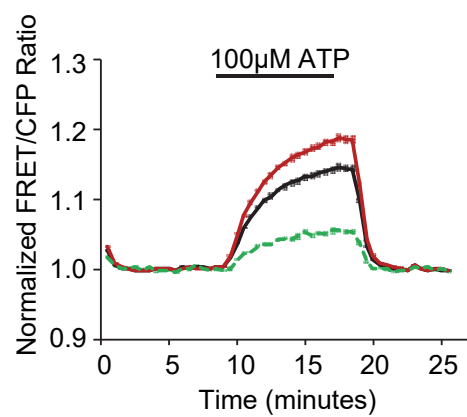

Supplement: S2 Fig — The whole cell average (black, middle trace) is reflective of the surface ecAT3.10 signal (n = 10 cells). Values and solid line traces are cell means, and errors and error bars are standard errors of the means. (PDF) [file pone.0187481.s006.pdf]

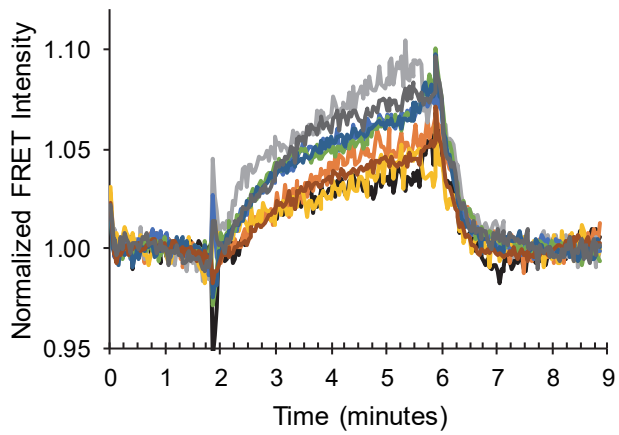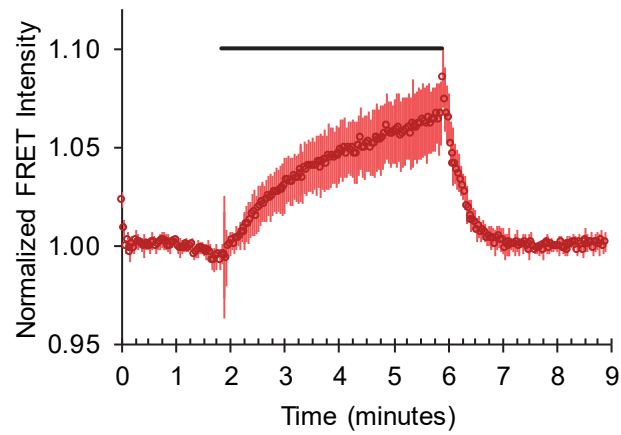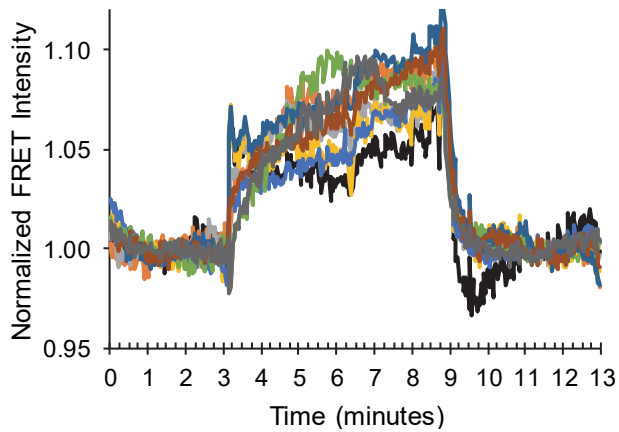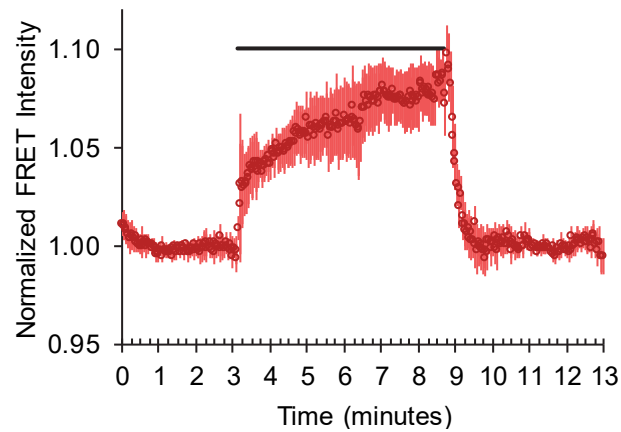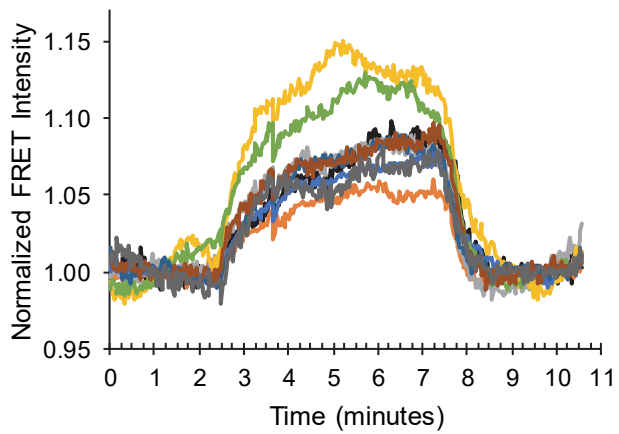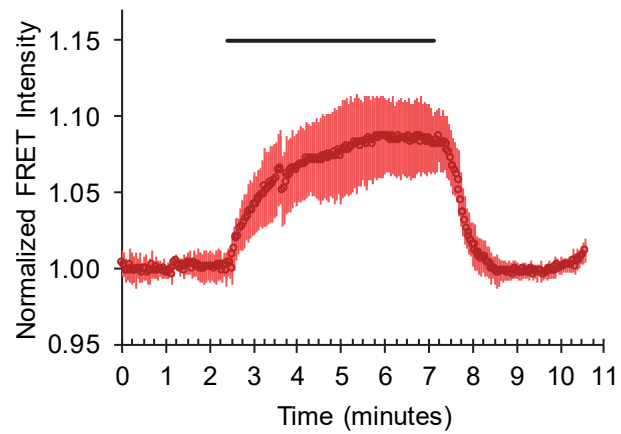

Supplement: S4 Fig — (A) The ecAT3.10 sensor responds within seconds to ATP addition. Individual cell responses (left panels) and mean (± standard error of the mean) response (right panels) for each of three experiments that are averaged in Fig 2. n = 9 cells per experiment. (PDF) [file pone.0187481.s008.pdf]

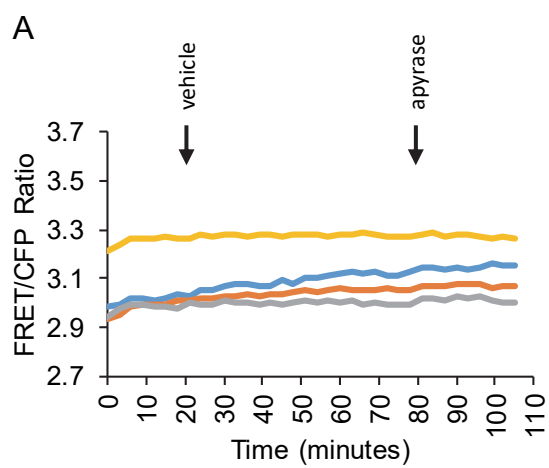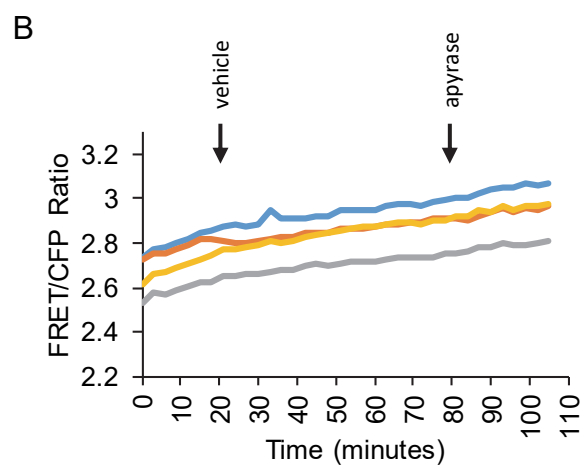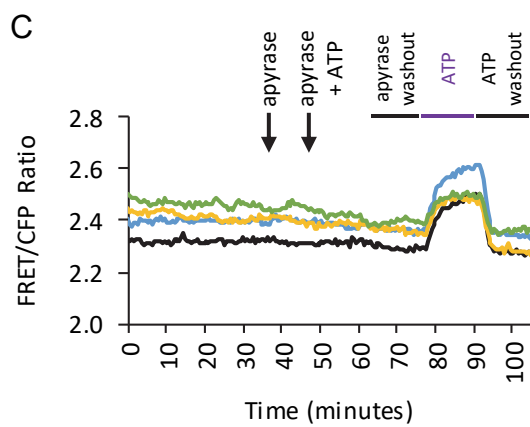

Supplement: S5 Fig — Ectonucleotidase inhibitor ARL67156 (100 μM) potentiates the ecAT3.10 ratio signal in response to 30μM ATP addition under static bath conditions in Neuro2A cells, but the (A) vehicle and (B) ARL67156 alone do not elicit a response. (C) Apyrase does not prevent ecAT3.10 response to extracellular ATP. 5 units/mL apyrase (high ATPase activity) was washed in at t = 37 minutes, and no change was observed. An addition of 100 μM ATP and 4.3 units/mL apyrase at t = 47 min also did not elicit a response. Cells were superfused with imaging solution to wash out apyrase and ATP at t = 62 min. At t = 77 min an addition of 100 μM ATP caused an increase in the ecAT3.10 ratio signal that could be reversed upon washout at t = 90 min, confirming function of the sensor following apyrase treatment. (PDF) [file pone.0187481.s009.pdf]

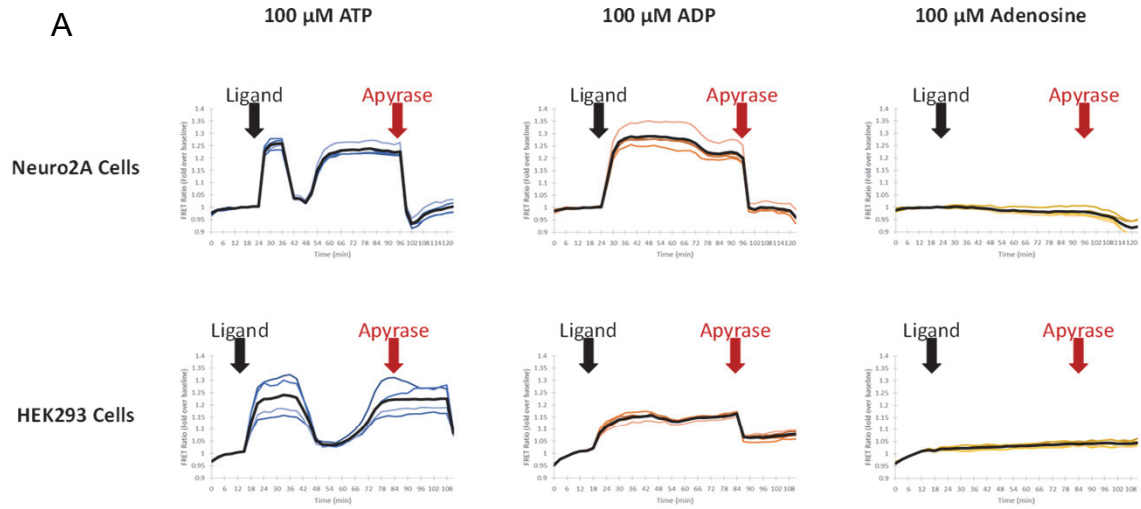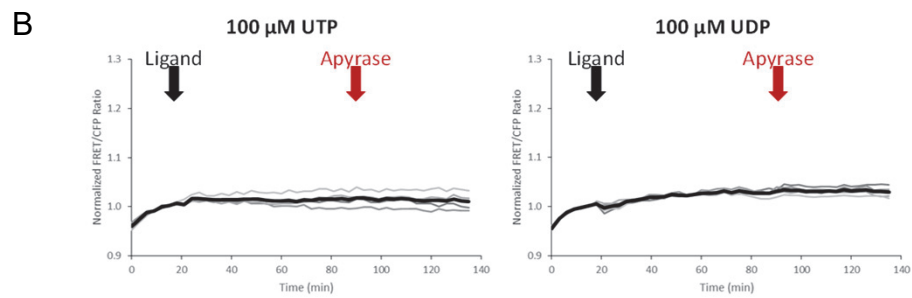

Supplement: S6 Fig — (A) ATP and ADP stimulate ATP release in ecAT3.10-expressing Neuro2A and HEK293 cells, but adenosine does not. (B) UTP, and UDP do not stimulate ATP release in Neuro2A cells. (PDF) [file pone.0187481.s010.pdf]

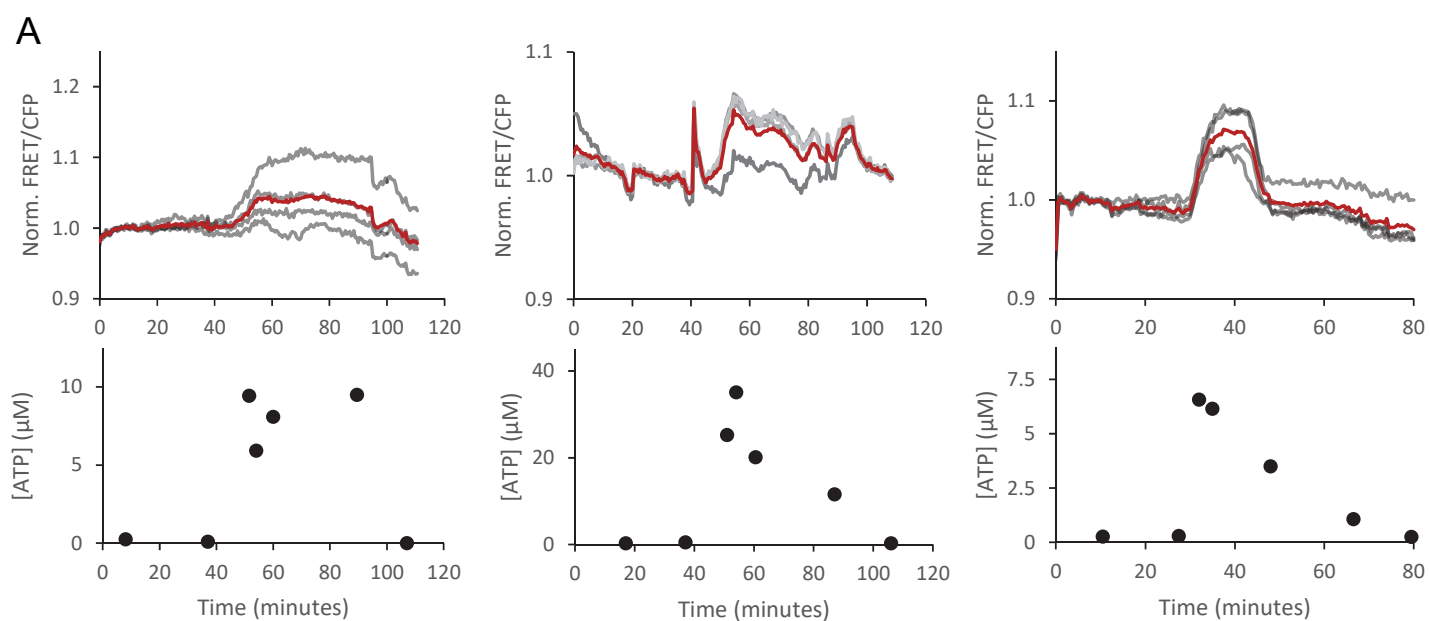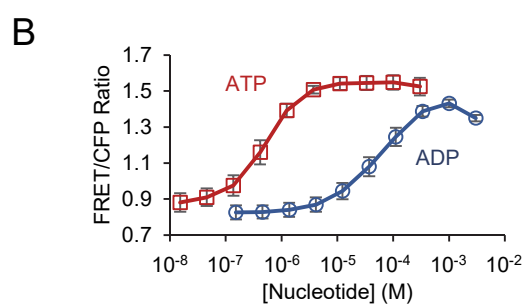

Supplement: S7 Fig — (A) Paired imaging and luciferase experiments. Top panels show ecAT3.10 imaging experiments with Neuro2A cells stimulated to release ATP by 100μM ADP, which was subsequently degraded by apyrase addition. Bottom panels show paired luciferase ATP end-point assays in which time points represent samples take from the bath from paired imaging experiments in the top panels. (B) In vitro dose-response of purified AT3.10 for ATP (Kapp = 0.52 ± 0.02 μM, mean ± sem, n = 14 experiments) and ADP (Kapp = 46 ± 5 μM, mean ± sem, n = 9 experiments) demonstrates a nearly 100-fold selectivity for ATP over ADP in solution. (PDF) [file pone.0187481.s011.pdf]

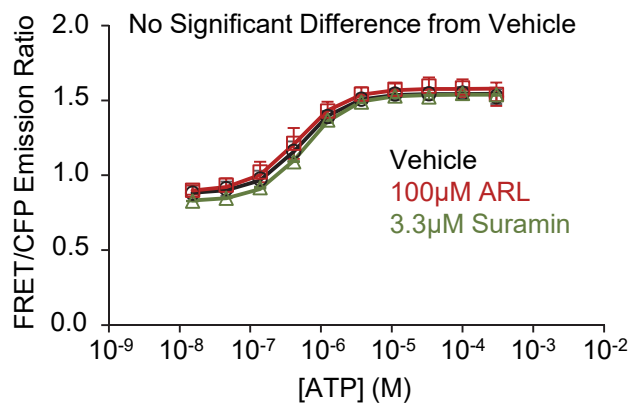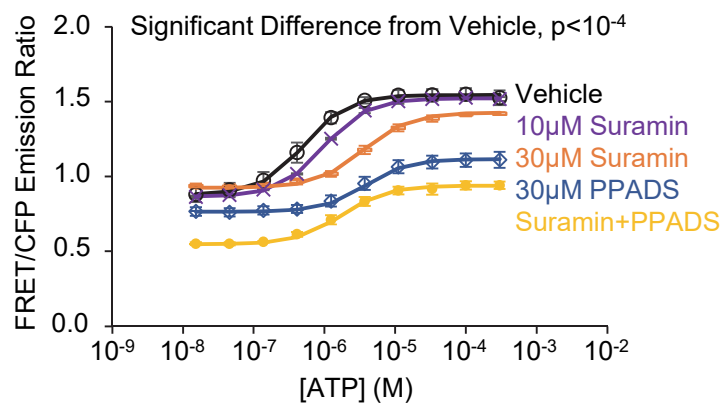

Supplement: S8 Fig — (Left) The presence of 100 μM ARL67156 or 3.3 μM suramin does not affect the sensor. (Right) At concentrations >10μM both suramin and PPADS significantly alter the sensor characteristics. (PDF) [file pone.0187481.s012.pdf]
