# Supplementary material for: Imaging extracellular ATP with a genetically-encoded, ratiometric fluorescent sensor
Source: PLoS One. 2017 Nov 9;12(11):e0187481. doi: 10.1371/journal.pone.0187481 (PMC5679667; doi:10.1371/journal.pone.0187481)
Supplement: S3 Fig — (A) Gel-shift assay in which gel fluorescence is imaged first followed by Coomassie staining of the same gel to visualize the fetuin controls. Treatment of cell lysates (lanes 4–5) or live cells (lanes 6–7) for 1 hour at 37°C with PNGaseF and O-glycosidase (from NEB in PBS supplemented with 1 mM CaCl2, 1 mM MgCl2, and 10 mM HEPES, pH 7.4; Hall et al. 2014 FEBS Open Bio. 4:892; Dellisanti et al. 2007 Nat. Neurosci. 10:953) does not cause a gel shift in the ecAT3.10 fluorescence band relative to untreated cells, indicating that the sensor is not glycosylated. Coomassie staining confirms that enzyme activity causes a gel shift for the non-fluorescent control protein fetuin (lanes 8–9). Samples in lanes 2–7 were left unboiled to preserve sensor fluorescence, and fluorescence was visualized with shortwave UV excitation. (C) Pre-treatment of cells for 1 hour at 37°C with PNGaseF, O-glycosidase, and neuramidase does not change the live-cell ATP dose response of ecAT3.10. (PDF) [file pone.0187481.s007.pdf]

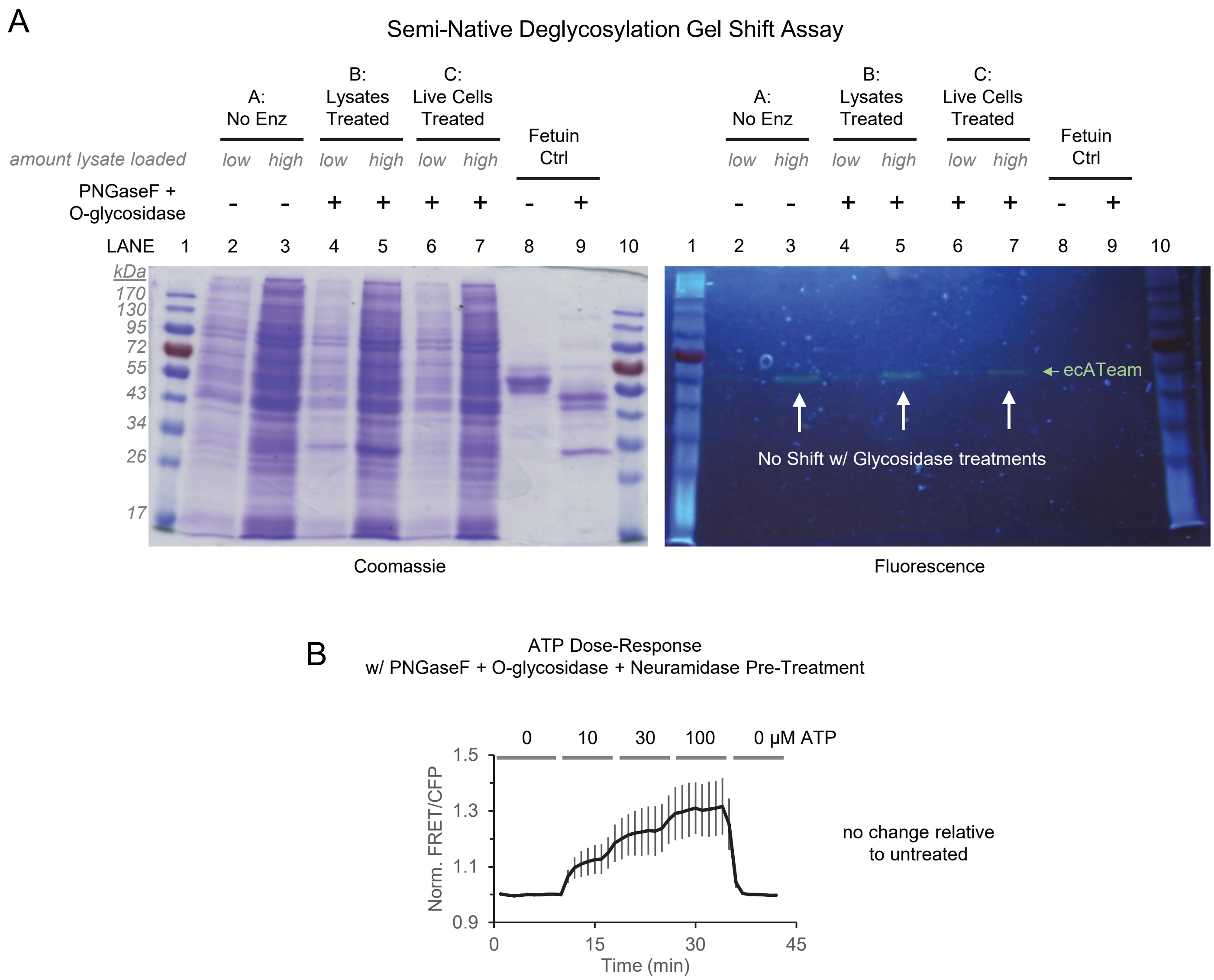
A
Semi-Native Deglycosylation Gel Shift Assay
A: No Enz
B: Lysates Treated
C: Live Cells Treated
Fetuin Ctrl
amount lysate loaded
low high low high low high
PNGaseF + O-glycosidase
- - + + + + - +
LANE 1 2 3 4 5 6 7 8 9 10
kDa
170
130
95
72
55
43
34
26
17
ecATeam
No Shift w/ Glycosidase treatments
Coomassie
Fluorescence
B
ATP Dose-Response
w/ PNGaseF + O-glycosidase + Neuramidase Pre-Treatment
0 10 30 100 0 μM ATP
Norm. FRET/CFP
1.5
1.3
1.1
0.9
0
15
30
45
Time (min)
no change relative to untreated
